# Comorbidity risk characteristics of rheumatoid arthritis in the context of depression-associated lipid metabolism

**DOI:** 10.3389/fimmu.2026.1806730

**Published:** 2026-05-26

**Authors:** Jiaru Liu, Cuitian Xiang, Qirui Zhou, Zhenzhu Ding, Jingrong Wang, Arong Li

**Affiliations:** 1 The Second Clinical College of Guangzhou University of Chinese Medicine, Guangzhou, China; 2 School of Automation, Guangdong University of Technology, Guangzhou, China; 3 State Key Laboratory of Traditional Chinese Medicine Syndrome, the Second Affiliated Hospital of Guangzhou University of Chinese Medicine, Guangzhou, China; 4 Chinese Medicine Guangdong Laboratory, Guangdong-Macao In-Depth Cooperation Zone in Hengqin, Zhuhai, China

**Keywords:** comorbidity, immune-inflammatory signature, lipid metabolism, major depressive disorder, neuron–synovium axis, rheumatoid arthritis

## Abstract

**Background:**

Rheumatoid arthritis (RA) and major depressive disorder (MDD) exhibit significant comorbidity, with shared pathological mechanisms such as inflammation and abnormal lipid metabolism. However, the specific molecular features and functional pathways linking the two diseases remain unclear.

**Methods:**

This study integrated public transcriptomic data from five MDD and five RA cohorts. Lipid metabolism-related module genes were screened in MDD cohorts and intersected with RA-upregulated differentially expressed genes to construct a candidate set. LASSO regression and machine learning (XGBoost, Random Forest) were used for feature selection and model construction. Functional exploration included protein-protein interaction network analysis, immune infiltration analysis, and multi-cohort validation. To experimentally test the proposed lipid–immune link and a potential neuron-to-synovium axis, we established an *in vitro* model. Hippocampal HT-22 neurons were subjected to combined glucocorticoid and saturated fatty acid stimulation to mimic MDD-associated stress–lipotoxicity. The conditioned medium (CM) from these neurons was then applied to human fibroblast-like synoviocytes (FLS). The inflammatory and functional responses of FLS were assessed through qPCR, ELISA, and Western blot.

**Results:**

A nine-gene lipid–immune signature (ANXA3, IL18, CD59, TNFSF13B, BMX, WASF1, SLC8A1, COMMD8, NXT2) was identified. It showed excellent discriminative performance in independent RA cohorts (AUC 0.86–1.00) but weaker performance in MDD cohorts (AUC 0.55–0.65). TNFSF13B, IL18, and CD59 were consistently identified as core hubs. The signature’s expression correlated significantly with immune cell infiltration in RA. Critically, our *in vitro* validation demonstrated that CM from lipid-stressed neurons directly activated FLS. This was evidenced by: (i) significant upregulation of CD59 mRNA and protein levels (via qPCR and Western blot); (ii) elevated secretion of key inflammatory mediators IL-6 and MMP3 (via ELISA); and (iii) enhanced migratory capacity of FLS. These data support a putative paracrine communication axis from stressed neurons to synovial cells, suggesting a role for lipid-mediated inflammatory reprogramming.

**Conclusion:**

This study identifies a conserved lipid–immune gene signature associated with the risk of MDD-RA comorbidity and provides multi-level validation encompassing bioinformatic prediction, clinical cohort correlation, and direct experimental evidence (qPCR, ELISA, WB). The signature may represent an immune-inflammatory state activated in a subset of MDD individuals that overlaps with RA pathology. Based on the hub genes, a “Lipid Metabolism – Membrane Microenvironment – Immune Inflammation” axis hypothesis is proposed. The experimentally demonstrated neuron–synovium paracrine axis, characterized by specific molecular changes (CD59, IL-6, MMP3), offers a novel and testable mechanistic conduit for cross-disease pathology, providing new molecular insights and directions for understanding psychiatric–immune comorbidity.

## 1 Introduction

### 1.1 Disease background

Rheumatoid arthritis (RA) is a chronic systemic autoimmune disease characterized primarily by persistent joint inflammation, pain, swelling, and progressive functional impairment. Prolonged inflammatory activity not only leads to irreversible joint destruction but also affects multiple organ systems, there by markedly reducing patients’ quality of life and work capacity.

Major depressive disorder (MDD) is a severe psychiatric condition defined by persistent low mood and loss of interest or pleasure, and it is widely recognized to be closely associated with chronic inflammatory processes (1). Clinically, MDD manifests as sustained sadness, anhedonia, excessive guilt, impaired concentration, and disturbances in sleep and appetite; in severe cases, suicidal ideation or behavior may also occur (2).

Notably, rheumatoid arthritis (RA) and major depressive disorder (MDD) primarily target the immune system and central nervous system, respectively. Despite this difference, they share key clinical features: both are chronic, driven by inflammation, and involve multiple organ systems. These common characteristics collectively contribute to a substantial burden on patients’ physical and psychological health.

### 1.2 Comorbidity between rheumatoid arthritis and major depressive disorder

MDD is one of the most common psychiatric comorbidities observed in patients with RA, and its high prevalence and clinical significance have been consistently demonstrated across multiple studies (3). In individuals with RA, persistent joint pain, swelling, and functional impairment not only substantially disrupt daily activities but also contribute to negative emotional states, including anxiety and depression, thereby further aggravating the overall psychological burden (4). Observational studies have shown that the prevalence of depression among patients with RA is significantly higher than that in the general population, suggesting a strong co-occurrence between RA and depressive disorders (5).

Beyond the increased susceptibility to depression in patients with RA, accumulating evidence from large-scale population-based cohort studies indicates that depression itself may represent an independent risk factor for the development of RA. For example, a nationwide study based on the Taiwan National Health Insurance Research Database reported a significantly higher incidence of RA among individuals with depression compared with non-depressed controls (6). Similarly, a prospective cohort study involving more than four million individuals in the United Kingdom demonstrated that patients with MDD had approximately a 38% increased risk of developing RA compared with those without MDD. Notably, this study also observed a relatively reduced risk among patients receiving antidepressant treatment, suggesting that improvement in mood or modulation of inflammatory activity may influence the onset of RA (7). Collectively, these findings indicate a potential bidirectional association between MDD and RA, rather than a unidirectional relationship driven solely by psychological reactions or secondary complications.

From a biological perspective, the association between depression and RA may be partly attributable to shared inflammation-driven mechanisms. In RA, several pro-inflammatory cytokines, including interleukin-1β IL-1β, tumor necrosis factor-α TNF-α, and interleukin-6 (IL-6), are chronically upregulated. These cytokines have also been repeatedly implicated as key mediators in the pathophysiology of depression within the framework of neuroinflammation research (3). Multiple high-quality reviews and meta-analyses have further emphasized that inflammatory signaling pathways play critical roles in the initiation and progression of both RA and MDD (8–10). Accordingly, the comorbidity between MDD and RA is unlikely to be coincidental, but may instead reflect a partial overlap in their underlying inflammatory networks.

Despite substantial evidence supporting the bidirectional epidemiological association and shared inflammatory features between MDD and RA, the fundamental nature of their comorbidity remains incompletely understood. In particular, whether common metabolic pathways exist, and how cross-disease molecular interactions contribute to the development of comorbidity at the systemic level, have yet to be fully elucidated. These unresolved questions highlight a critical gap in current knowledge and underscore the need for further investigation into the shared pathological basis linking MDD and RA.

### 1.3 Lipid metabolism as a shared mechanism linking MDD and RA

In studies investigating lipid-related alterations in MDD, accumulating evidence indicates that disruption of lipid homeostasis occurs across multiple stages of disease onset and progression. Compared with healthy controls, patients with MDD exhibit significant abnormalities in triglycerides, lysophospholipids, phospholipids, and various unsaturated sphingolipids, pointing to dysregulation of glycerolipid (GL), glycerophospholipid (GP), and sphingolipid (SP) metabolic pathways. These pathways play essential roles in maintaining cellular membrane integrity, regulating transmembrane signaling, and modulating neuroinflammatory responses (11).

Findings from large-scale population studies further support this trend. Plasma lipidomic analyses have demonstrated close associations between MDD and multiple lipid classes, including unsaturated sphingolipids, phosphatidylcholine (PC), phosphatidylethanolamine (PE), phosphatidylinositol (PI), and lysophosphatidylcholine (LPC) (12, 13). Moreover, an untargeted lipidomics study comparing patients with MDD n = 60 and healthy controls n = 60 identified significant alterations in a broad spectrum of lipids, including LPC, lysophosphatidylethanolamine (LPE), PC, PE, PI, ether-linked phosphatidylcholine (PC-O), diacylglycerols (DG), and triacylglycerols (TG). Notably, levels of several lipid species were positively correlated with depression severity, suggesting that lipid metabolic disturbances may be closely associated with disease phenotypes (14). Collectively, these findings highlight lipid metabolism–related alterations as a prominent molecular feature of MDD.

Similarly, substantial lipid metabolic remodeling has been observed in RA. Lipidomic analyses of serum and synovial fluid across different disease stages, including the preclinical phase, active disease, and remission, have revealed that changes in lysophosphatidylcholine, phosphatidylcholine, ether-linked phosphatidylethanolamine, and multiple sphingolipid subclasses are closely associated with RA disease activity (15). In addition, a comparative lipidomics study between patients with RA and healthy individuals identified 26 differential serum lipids and three differential urinary lipids, encompassing LPC, ether-linked LPC (LPC-O), PC, plasmalogen phosphatidylethanolamine (PE-P), sphingomyelins (SM), and acylcarnitines (CAR). These lipid species were proposed as potential biomarkers for distinguishing seropositive and seronegative RA (16).

Taken together, both MDD and RA exhibit highly similar patterns of lipid abnormalities, particularly involving key lipid species such as lysophospholipids (LPC/LPE), phosphatidylcholine (PC), and sphingolipids (SM/ceramides), as well as their associated metabolic pathways. These convergent lipidomic signatures suggest that lipid metabolism–related alterations may constitute an important biological framework and mechanistic bridge linking MDD and RA, thereby providing a theoretical basis for further investigation into the risk and molecular features of their comorbidity.

### 1.4 Knowledge gaps in understanding lipid-mediated comorbidity between MDD and RA

Although existing studies have revealed a potential bidirectional association between MDD and RA from epidemiological and inflammatory biology perspectives, the interactive mechanisms linking these two diseases at the level of lipid metabolism remain poorly understood. To date, most lipidomics studies have focused on single-disease contexts, primarily characterizing lipid abnormalities within MDD or RA independently, while systematic cross-disease comparisons of lipid metabolic features are largely lacking. Moreover, despite increasing evidence for lipid metabolic dysregulation in MDD, few studies have attempted to link these alterations to the risk of RA development, and molecular evidence explaining a potential MDD-to-RA comorbidity pathway from a lipid metabolism perspective remains scarce.

At the methodological level, existing research has largely relied on correlation-based analyses or single-cohort datasets, with limited use of integrative, cross-cohort, and cross-disease analytical strategies. This limitation hampers the identification of robust and reproducible lipid metabolic features with consistent relevance across diseases, particularly within the complex and highly interconnected lipid metabolic network. Consequently, molecular biomarkers capable of predicting RA comorbidity risk based on MDD-associated lipid metabolic signatures are still lacking, as is a systematic computational framework to elucidate how lipid metabolism may function as a biological link between MDD and RA.

Collectively, these gaps highlight an urgent need for integrative strategies that combine MDD-related lipid metabolic abnormalities with multi-cohort RA datasets. Such approaches are essential to identify cross-disease, pathway-spanning comorbidity risk features and to bridge the current knowledge gap in understanding the shared lipid-mediated mechanisms underlying MDD–RA comorbidity.

### 1.5 Study aims and innovation

Based on the above, this study aimed to leverage lipid metabolic dysregulation as a functional context to identify and functionally validate molecular features linking MDD to RA comorbidity. Specifically, we hypothesized that lipid metabolism–associated transcriptional programs in MDD might be particularly relevant to RA pathogenesis. To test this, we first identified gene co-expression modules tightly correlated with lipid metabolic activity in MDD cohorts. We then intersected these module genes with RA-dysregulated genes to derive a shared molecular signature, which was further refined using machine learning and network analysis. Finally, to move beyond computational association and assess biological relevance, we experimentally validated the functional impact of this lipid–immune interface using an *in vitro* neuron–synovium communication model.

The innovation of this study lies in its integrative strategy that couples a lipid-centric discovery filter with direct experimental validation. By first identifying lipid-correlated co-expression modules in MDD and intersecting them with RA transcriptomic alterations, we uncovered shared molecular networks at the lipid–immune interface. Crucially, we then functionally tested this interface by stimulating neurons under lipid-stress conditions and assessing their paracrine effects on synovial cells. This approach shifts the focus not only from individual molecules to contextualized networks but also from computational prediction to mechanism-supported biological relevance, providing a more robust framework for understanding MDD–RA comorbidity.

## 2 Materials and methods

### 2.1 Data acquisition and preprocessing

**Figure 1:**
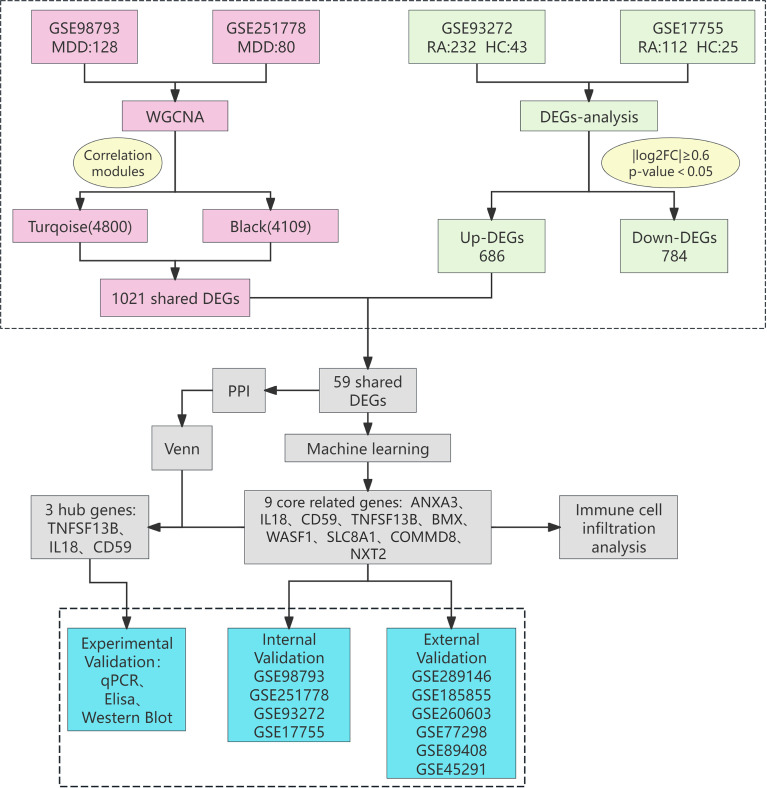
Study flowchart. RA, rheumatoid arthritis; MDD, major depressive disorder; GSE, Gene Expression Omnibus series; WGCNA, weighted gene co-expression network analysis; DEGs, differentially expressed genes; GO, Gene Ontology; KEGG, Kyoto Encyclopedia of Genes and Genomes; PPI network, protein-protein interaction network; ROC curve, receiver operating characteristic curve.

All transcriptomic datasets used in this study were obtained from the Gene Expression Omnibus (GEO) database and downloaded in MINIML format (corresponding references are provided in the main text). The overall study workflow is illustrated in **Figure 1**. A total of ten independent cohorts were included, comprising five MDD cohorts and five RA cohorts. Detailed information on dataset platforms, sample grouping, and accession numbers is summarized in **Table 1**.

**Table 1:** Datasets.

No.	Disease	Geo	Patients (n)	Healthy controls (n)	Role in analysis
1	MDD	GSE98793	128	64	Feature construction and internal validation
2	MDD	GSE251778	80	89	
3	RA	GSE93272	232	43	Feature selection, model construction, and internal validation
4	RA	GSE17755	112	45	
5	MDD	GSE289146	229	70	External validation
6	MDD	GSE185855	47	22	
7	MDD	GSE260603	240	33	
8	RA	GSE77298	16	7	
9	RA	GSE89408	95	28	
10	RA	GSE45291	493	20	

All expression matrices were processed and analyzed in the R environment. For datasets containing untransformed raw expression values, log_2_ transformation was performed before downstream analysis, whereas datasets already log-transformed were used directly. Each dataset was subsequently normalized using the normalizeBetweenArrays function (limma package) to ensure cross-sample comparability and reduce technical variation. For analyses requiring the integration of multiple datasets (specifically the RA cohorts GSE93272 and GSE17755), batch effects across cohorts were corrected using the ComBat function (sva package). Principal component analysis (PCA) was performed before and after batch correction to evaluate whether technical variation across datasets was reduced while preserving biological differences between disease and control groups. As further supported by the additional evidence provided below, this preprocessing pipeline provided a robust foundation for subsequent differential expression, WGCNA, and machine learning analyses by minimizing the risk of cohort-specific bias. To manage technical and biological heterogeneity across the ten GEO cohorts, we applied standardized normalization and batch-effect correction. This approach aimed to identify a core molecular signal that remains stable despite the technical variation typically found in multi-center datasets.

### 2.2 Construction of a lipid metabolism-related gene set and calculation of ssGSEA scores

To systematically assess sample-level lipid metabolic activity, we first compiled a candidate gene set potentially related to lipid metabolism from the GeneCards database using the keyword “lipid metabolism.” Given that lipid metabolism involves multiple regulatory layers—including biosynthesis, transport, signaling, and crosstalk with immune and inflammatory pathways—and considering the subsequent need for co-expression network analysis and machine learning–based feature selection, we adopted an inclusive strategy at this initial stage. Specifically, a GeneCards relevance score > 30 was selected as the threshold to prioritize genes with established lipid associations while maintaining sufficient breadth to capture emerging regulatory signals, thereby ensuring the initial candidate pool encompasses multi-layered regulatory activity and minimizing false-negative losses during cross-disease integration.

It should be emphasized that this GeneCards-derived set was not intended as a definitive “lipid metabolism–specific” gene list. Instead, it served as a hypothesis-generating pool to broadly capture transcriptional activity associated with lipid processes. This candidate pool was subsequently refined and functionally validated through a series of analytical steps—including ssGSEA scoring, WGCNA module identification, differential expression analysis, and machine learning–based feature selection—thereby reducing potential bias from single-source annotations.

Based on this lipid metabolism–related gene set, single-sample gene set enrichment analysis (ssGSEA) was performed in two MDD cohorts (GSE98793 and GSE251778) to calculate a lipid metabolism score for each sample. Samples were then dichotomized into high- and low-lipid metabolism groups using the median score as the cutoff, establishing a phenotypic stratification for downstream co-expression module detection and comparative analyses.

### 2.3 WGCNA construction and identification of lipid-related modules

To explore co-expression modules associated with lipid metabolism scores, weighted gene co-expression network analysis (WGCNA) was performed in the specified cohorts (17). The procedure was as follows:

1. The top 10,000 genes ranked by standard deviation in the expression matrix were selected for network construction.
2. Low-quality genes and samples were removed using the goodSamplesGenes function.
3. An appropriate soft-thresholding power (β) was chosen according to the scale-free topology criterion to construct the adjacency matrix, which was subsequently transformed into a topological overlap matrix (TOM).
4. Hierarchical clustering combined with dynamic tree cutting was applied to identify modules, and module eigengenes (MEs) were calculated for each module.
5. Pearson correlation coefficients between each module and the lipid metabolism score were computed. In each cohort, the module showing the strongest correlation with the lipid metabolism score was defined as the key module.

### 2.4 Differential expression analysis and candidate comorbidity gene selection

Within the RA combined cohorts, differential expression analysis between patients and healthy controls was conducted using the limma R package. Differentially expressed genes (DEGs) were identified using the thresholds: P < 0.05 and |log2 fold change| > 0.6 (equivalent to ~1.5-fold change). This relaxed threshold was applied to accommodate inter-cohort heterogeneity across the five independent RA datasets while identifying robust molecular perturbations that consistently drive disease pathology.

Considering the positive correlation between lipid metabolism–related modules and the lipid metabolism score, and focusing on activated signals relevant to RA progression, downstream intersection analyses were performed using RA upregulated DEGs. Specifically, RA upregulated DEGs were intersected with genes from lipid-related WGCNA modules to generate a candidate gene set potentially associated with MDD–RA comorbidity.

### 2.5 Machine learning–based selection of key feature genes and model validation

To refine the candidate gene set and identify robust feature genes for RA classification, a sequential machine learning pipeline was implemented, comprising feature selection, model training, interpretation, and validation. Unless otherwise specified, all XGBoost analyses in this study were performed using the same parameter settings described below.

1. Feature selection via LASSO regression: The 59 intersecting RA-upregulated and MDD lipid module genes were further screened using LASSO logistic regression implemented in the glmnet package. The gene expression matrix was used as the predictor matrix, and the binary disease status was used as the response variable. A binomial model with an L1 penalty was fitted by setting family = “binomial” and alpha = 1. A fixed random seed was used to ensure reproducibility, and five-fold cross-validation was performed during model fitting to assess the regularization path. To obtain a parsimonious and interpretable feature set, we selected the penalty parameter whose associated number of nonzero coefficients was closest to the predefined target number of ten predictors, considering only models with at least one retained feature. The genes with nonzero coefficients under this lambda value were defined as LASSO-selected feature genes. Finally, nine genes *(ANXA3*, *IL18*, *CD59*, *TNFSF13B*, *BMX*, *WASF1*, *SLC8A1*, *COMMD8*, and *NXT2*) were retained for downstream classification model construction and validation.
2. Model training and feature importance assessment: Using the nine LASSO-selected feature genes, we constructed RF and XGBoost classifiers to distinguish RA patients from healthy controls. The XGBoost model was trained with objective = “binary:logistic”, nrounds = 200, max_depth = 3, eta = 0.1, subsample = 0.8, colsample_bytree = 0.8, and eval_metric = “logloss”. The RF model was implemented using the ranger package with probability = TRUE, num.trees = 1000, mtry = 3, and min.node.size = 5. Feature importance scores from both models were used to evaluate the relative contribution of each gene to RA classification.
3. Model interpretation with SHAP: To enhance interpretability, SHapley Additive exPlanations (SHAP) analysis was performed, primarily based on the XGBoost model results, to quantify and visualize the direction and magnitude of each gene’s contribution to the prediction.
4. Model performance evaluation: The performance of the classification models (particularly the XGBoost model based on the nine-gene signature) was rigorously assessed. Internally, five-fold cross-validation was conducted on the training cohorts to ensure stability and mitigate overfitting. Externally, the generalizability of the model was further validated by applying it to independent validation cohorts.

### 2.6 PPI network construction and hub gene identification

Candidate intersection genes were input into the STRING database to construct a protein–protein interaction (PPI) network, which was visualized and analyzed topologically in Cytoscape. Node ranking was performed using the igraph R package with four algorithms: Degree, Closeness, Betweenness, and Shortest Path. Hub genes were defined based on consensus across multiple ranking algorithms.

Specifically, in this study, “1021” refers to the intersection of lipid metabolism–related key module genes from the two MDD cohorts, representing the common lipid module genes, while “1434” denotes the differentially expressed genes identified in the combined RA cohorts (RA-DEGs). Subsequently, RA upregulated DEGs were intersected with the 1021 lipid module common genes, yielding 59 candidate comorbidity-related genes. These were further refined through machine learning analyses to produce a final nine-gene feature set.

### 2.7 Internal and external validation

The identified nine-gene signature was further validated using both internal and external datasets. Internal validation was first performed in four cohorts, including GSE98793, GSE251778, GSE93272, and GSE17755. An XGBoost classifier was constructed, and five-fold cross-validation was applied to evaluate model stability and generalizability.Receiver operating characteristic (ROC) curves were generated, and the area under the curve (AUC) was calculated to assess the discriminative performance of the model.

Subsequently, external validation was conducted in six independent cohorts, namely GSE289146, GSE185855, GSE260603, GSE77298, GSE89408, and GSE45291, to further evaluate the robustness and generalizability of the nine-gene signature across different populations and platforms.

### 2.8 Immune cell infiltration analysis

To evaluate the relationship between the nine feature genes and the immune microenvironment, immune cell infiltration analysis was performed in the RA cohorts (GSE93272 and GSE17755) using the CIBERSORT algorithm. CIBERSORT applies the LM22 signature matrix to deconvolute bulk transcriptomic data and estimate the relative proportions of 22 immune cell subtypes, including B cells, T cells, monocytes, macrophages, and neutrophils.

Spearman correlation analysis was subsequently conducted to assess the associations between the expression levels of the nine feature genes and the infiltration proportions of different immune cell types. Corresponding P values were calculated. The results were visualized using bubble plots, in which dot size represents statistical significance (–log10 P value) and color indicates the direction and strength of correlation.

In addition, to explore the functional relevance of the nine genes in MDD cohorts, their correlations with lipid metabolism–related, immune-related, neural, and stress-response pathways were analyzed in GSE98793 and GSE251778. These results were visualized using heatmaps.

### 2.9 *In vitro* validation of the neuron–synovium axis

**Figure 2:**
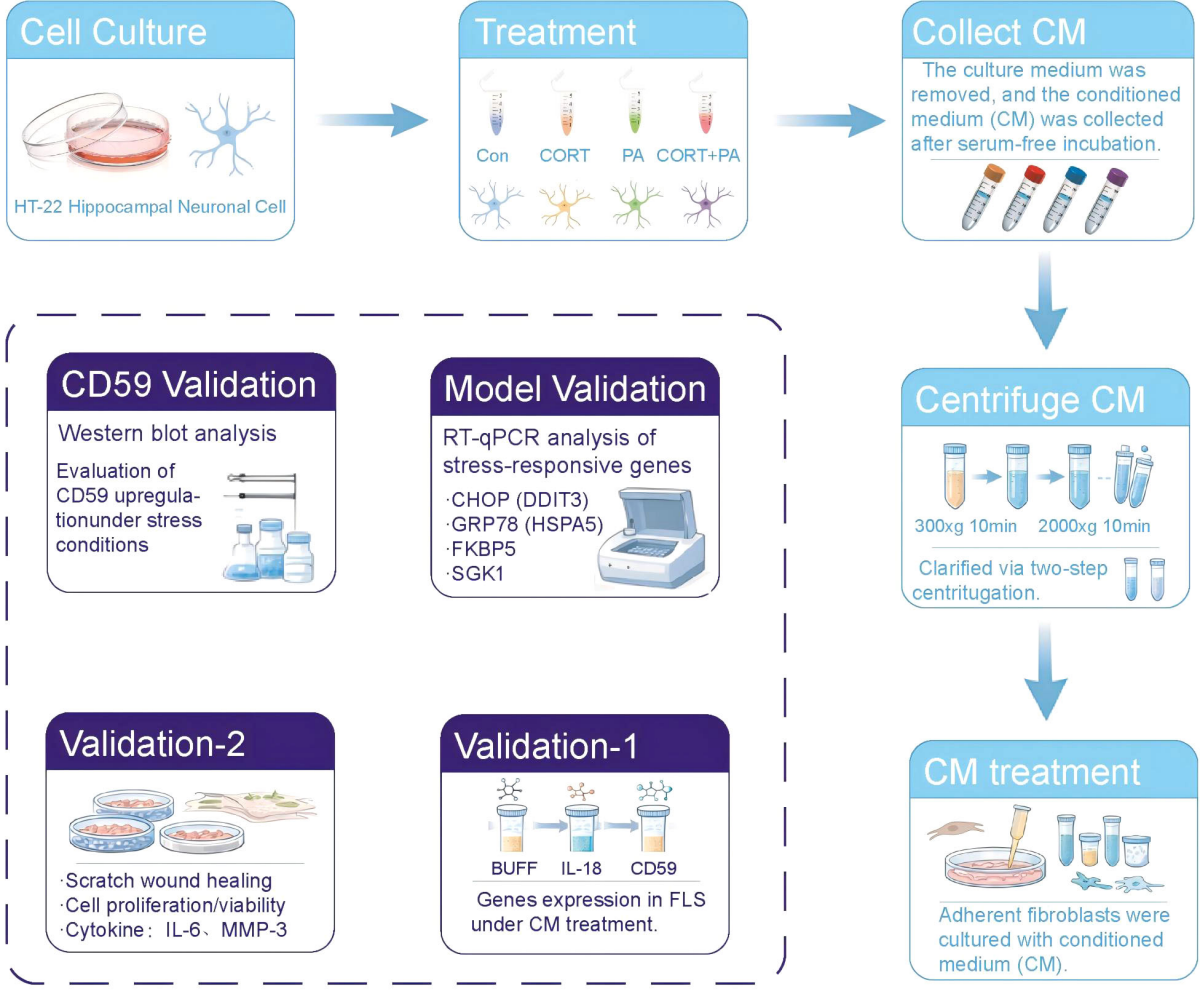
Experimental workflow for HT-22 stress model establishment and CM-mediated assays. HT-22 cells were treated with Ctrl, CORT, PA, or CORT + PA. Conditioned medium (CM) was collected after serum-free incubation and clarified by sequential centrifugation. Stress model validation was performed by qPCR and Western blot. Clarified CM was then applied to fibroblast-like synoviocytes (FLS) for functional analyses.

(A schematic flowchart of the experimental design is provided in **Figure 2**).

#### 2.9.1 Cell culture and treatment

Hippocampal neuron and fibroblast-like synoviocyte culture models were established for subsequent experiments. The mouse hippocampal neuronal cell line HT-22 was obtained from Jennio Biotech Co., Ltd (Guangzhou, Guangdong, China; Cat. No. JNO-M0087). Cells were maintained in Dulbecco’s Modified Eagle Medium (DMEM) supplemented with 10% fetal bovine serum (FBS) and 1% penicillin/streptomycin at 37°C in a humidified incubator with 5% CO_2_ (18). To mimic major depressive disorder (MDD)-associated stress and lipid metabolic dysregulation, HT-22 cells were exposed to different treatments: control cells received vehicle only (0.1% ethanol and fatty acid-free BSA), the CORT group was treated with 200µM corticosterone for 24h (19), the PA group received 0.25mM palmitic acid complexed with fatty acid-free BSA for 24h (20), and the combined CORT+PA group was co-treated with both agents for 24h.

To ensure the neural secretome was derived from viable neurons, the safety of the 200 µM CORT and 0.25 mM PA combination was validated using CCK-8 assays. After 24 hours of treatment, HT-22 cell viability remained above 90% across all groups with no significant difference compared to the control (*P* > 0.05) (**Supplementary Figure 1**). Additionally, to standardize the Conditioned medium (CM) collection, cells were seeded at a uniform initial density. Prior to CM collection, cell numbers were verified in parallel wells (**Supplementary Figure 2**), and phase-contrast microscopy was employed to confirm a comparable confluence (approximately 85%–90%) and typical neuronal morphology across all groups (**Supplementary Figure 3**). These rigorous controls ensure that the subsequent functional effects observed in synoviocytes were driven by specific paracrine signaling rather than variations in cell density or acute cytotoxicity. Human fibroblast-like synoviocytes (HFLS) were purchased from Sigma-Aldrich (Merck, Darmstadt, Germany), originally supplied by Cell Applications, Inc. (San Diego, CA, USA; Cat. No. 408-05A). Cells were cultured in RPMI-1640 medium containing 10% FBS and 1% penicillin/streptomycin under the same incubation conditions and were used for downstream experiments. Cells between passages 3 and 6 were used for all experiments.

#### 2.9.2 Conditioned medium collection and preparation

After 24h of treatment, HT-22 cells were gently washed twice with pre-warmed phosphate-buffered saline (PBS) to remove residual stimuli and serum components. The medium was then replaced with serum-free basal DMEM, and the cells were incubated for an additional 24h to allow the accumulation of paracrine factors. The 24-hour duration for serum-free incubation was selected based on pilot experiments evaluating a time gradient (12, 24, and 36 hours), which identified 24 hours as the optimal duration for accumulating a concentrated secretome without compromising neuronal health. CCK-8 assays demonstrated that HT-22 cell viability under serum-free conditions remained above 85% at 24 hours, with no statistically significant difference compared to cells maintained in serum-containing medium (*P* > 0.05) (**Supplementary Figure 4**). Morphological monitoring via phase-contrast microscopy further confirmed that HT-22 cells across all groups maintained typical neuronal structures and intact synaptic networks during the incubation (**Supplementary Figure 5**). By standardizing the serum-free protocol across all treatment and control groups, the metabolic impact of nutrient deprivation was controlled as a baseline constant, ensuring that the observed functional effects on synoviocytes were specifically driven by drug-induced paracrine signals rather than serum-deprivation artifacts. The resulting supernatant was collected as conditioned medium (CM). To ensure the removal of all cellular components and obtain a particle-free CM suitable for downstream functional assays, the CM was processed sequentially: (1) centrifugation at 300×g for 10 min to pellet any floating cells; (2) transfer of the supernatant to a new tube, followed by centrifugation at 2,000×g for 10 min to eliminate cell debris; and (3) filtration through a 0.22μM sterile filter to remove any remaining particulates or potential microbial contaminants. The clarified and sterile-filtered CM was aliquoted and stored at −80°C to preserve the integrity of secreted proteins and bioactive molecules until use.

#### 2.9.3 Validation of HT-22 cellular models

To confirm successful modeling, HT-22 cells were harvested after treatment for RNA extraction. Quantitative real-time PCR (qPCR) was performed using the following stress- and lipotoxicity-related markers:CORT model: FKBP5 and SGK1 (glucocorticoid receptor signaling) (21). PA model: CHOP and GRP78 (endoplasmic reticulum stress) (22). GAPDH served as the internal control. Primer sequences are listed in **Supplementary Table 1**.

#### 2.9.4 Functional assays on FLS

FLS were seeded in culture plates according to the specific assay format and allowed to adhere overnight. The culture medium was then replaced with CM derived from the four HT-22 treatment groups (Ctrl, CORT, PA, CORT+PA) (23–25) for functional stimulation.

After 24 h of CM stimulation, the following assays were performed:

Scratch wound healing assay: FLS were cultured to full confluence in 6-well plates. A uniform scratch wound was created across the monolayer using a sterile 200 μL pipette tip (26). After washing with PBS to remove detached cells, the wells were replenished with the respective CM. Migration was monitored by capturing images at 0 h and 24 h post-scratch using an inverted microscope. The migratory capacity of FLS was quantified by measuring the percentage of wound closure using ImageJ software.

Cell proliferation/viability assay: FLS were seeded in 96-well plates at a density of 5×10³ cells/well and treated with CM for 24 h. Cellular metabolic activity, as an indicator of proliferation/viability, was assessed using a Cell Counting Kit-8 (CCK-8, Dojindo) according to the manufacturer’s protocol (27). The absorbance was measured at 450 nm using a microplate reader.

Cytokine and protease secretion analysis: Following 24 h of CM stimulation, the supernatant from FLS cultures was collected and centrifuged to remove cellular debris. The concentrations of interleukin-6 (IL-6) and matrix metalloproteinase-3 (MMP-3) in the clarified supernatant were quantified using commercially available enzyme-linked immunosorbent assay (ELISA) kits (R&D Systems) according to the manufacturer’s standard protocols (28, 29).

#### 2.9.5 Molecular validation of hub gene expression in FLS

Following CM stimulation, total RNA was extracted from FLS using TRIzol reagent (Invitrogen). Reverse transcription was performed with the PrimeScript RT reagent kit (Takara). qPCR was carried out to measure the mRNA expression of the three core hub genes: IL18, TNFSF13B (BAFF), and CD59. GAPDH was used as the endogenous control. Primer sequences are provided in **Supplementary Table 1**.

For protein-level validation, total protein was extracted from FLS using RIPA lysis buffer. Western blotting was performed with primary antibodies against CD59 (1:1000, Abcam) and β-actin (1:5000, Cell Signaling Technology), followed by incubation with appropriate HRP-conjugated secondary antibodies. Protein bands were visualized using an enhanced chemiluminescence detection system.

#### 2.9.6 Statistical analysis

All *in vitro* experiments were performed in at least three independent replicates. Data are presented as mean ± standard deviation (SD). Differences between multiple groups were analyzed by one-way analysis of variance (ANOVA) followed by Tukey’s *post hoc* test. A p-value < 0.05 was considered statistically significant. All statistical analyses were conducted using GraphPad Prism 9.0.

## 3 Results

### 3.1 Identification of lipid metabolism–related modules in MDD cohorts

**Figure 3:**
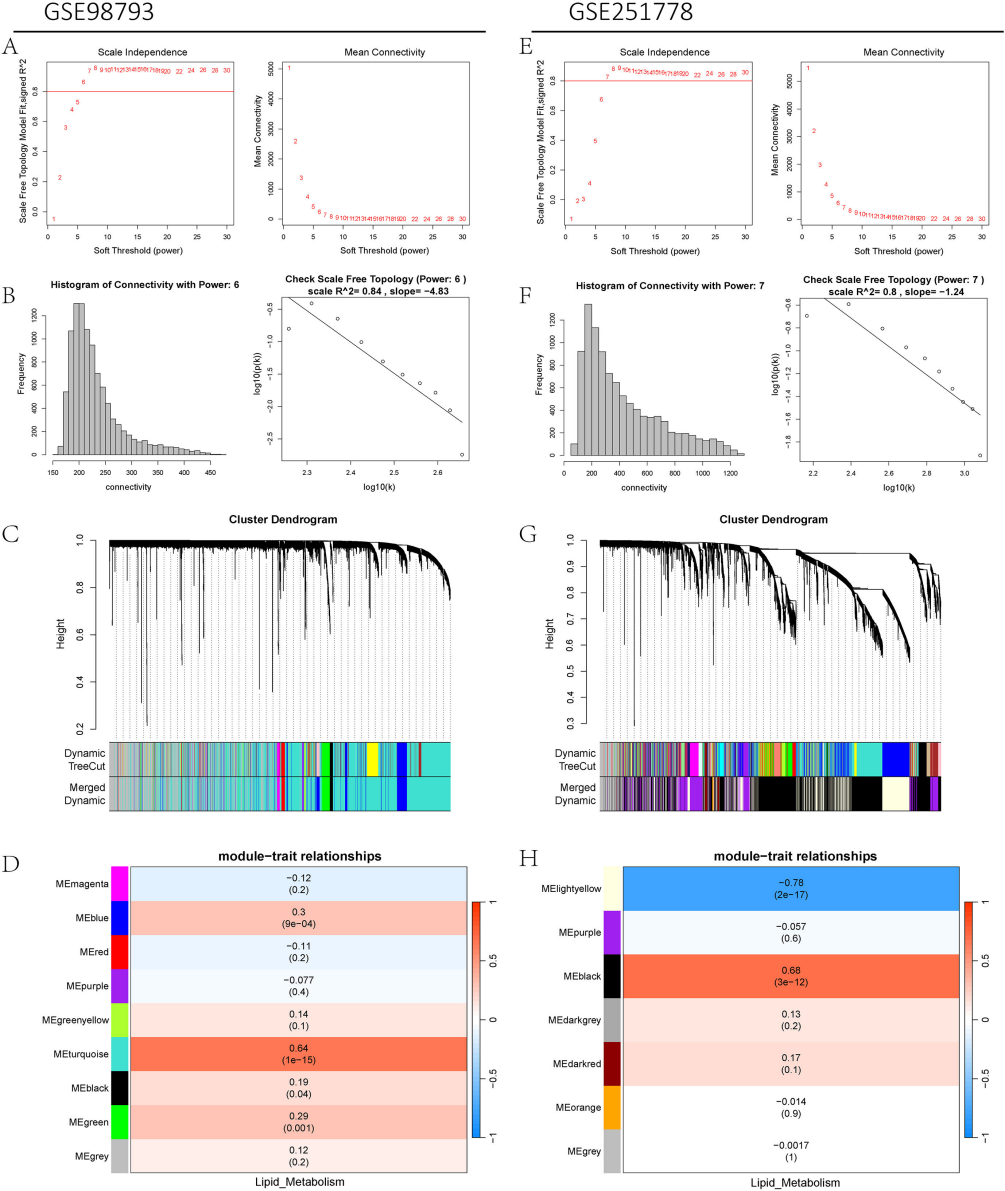
Identification of key regulators of lipid metabolism in MDD. **(A, B)** The soft-thresholding power was determined to be 6. **(C)** Construction of a weighted co-expression network utilizing the selected power. **(D)** Heatmap displaying the associations between modules and clinical traits (lipid metabolism score). **(E, F)** The soft-thresholding power for the second MDD cohort was established at 7. **(G)** Weighted co-expression network modeled using the selected power. **(H)** Heatmap illustrating the correlations between modules and clinical traits.

Following the calculation of lipid metabolism scores using ssGSEA, WGCNA was performed in the GSE98793 cohort. A soft-thresholding power of 6 was selected (**Figure 3A**), resulting in the identification of nine co-expression modules (**Figure 3B**). Module–trait correlation analysis indicated that the turquoise module exhibited the strongest association with the lipid metabolism score (r = 0.64; **Figure 3C**).

Similarly, WGCNA was applied to the GSE251778 cohort with a soft-thresholding power of 7, identifying seven modules. Among these, the black module showed the highest correlation with the lipid metabolism score (r = 0.68; **Figures 3D–F**).

Genes from the key modules identified in both cohorts were intersected to generate a set of MDD lipid metabolism–related genes for subsequent analyses.

### 3.2 PPI network and functional enrichment analysis of lipid metabolism–related genes

**Figure 4:**
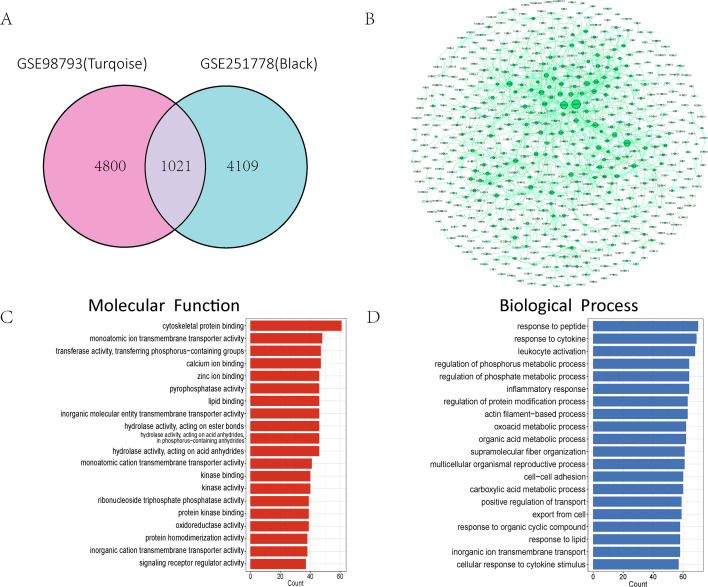
Functional analysis of lipid metabolism-related genes. **(A)** Screening for key lipid metabolism regulatory genes within the identified modules. **(B)** Protein-protein interaction (PPI) network of lipid metabolism regulatory genes. **(C, D)** GO enrichment analysis.

The key module genes significantly associated with lipid metabolism scores in GSE98793 were intersected with the corresponding key module genes from GSE251778, yielding a total of 1,021 shared lipid metabolism–related genes (hereafter referred to as lipid-module overlap genes; **Figure 4A**). A protein–protein interaction (PPI) network for these genes was constructed using the STRING database, which revealed a tightly interconnected network, suggesting potential functional coordination among these genes (**Figure 4B**).

Gene Ontology (GO) enrichment analysis was performed on the lipid-module overlap genes. Molecular function (MF) analysis indicated significant enrichment in cytoskeletal protein binding, ion binding (e.g., calcium and zinc ions), lipid binding, kinase activity, and transmembrane transport, as well as diverse hydrolase and oxidoreductase activities (**Figure 4C**).

Biological process (BP) enrichment analysis revealed that these genes were predominantly associated with immune and inflammatory responses, including cellular responses to cytokine and peptide stimuli, leukocyte activation, and inflammatory processes. Additional enriched processes included phosphate metabolic regulation, lipid response, cell–cell adhesion, cytoskeletal remodeling, and material transport (**Figure 4D**).

Collectively, these results suggest that lipid metabolism–related module genes may act synergistically in disease pathogenesis by modulating both immune–inflammatory responses and metabolic regulatory processes.

### 3.3 Differential expression analysis in RA combined cohorts and identification of candidate comorbidity genes

**Figure 5:**
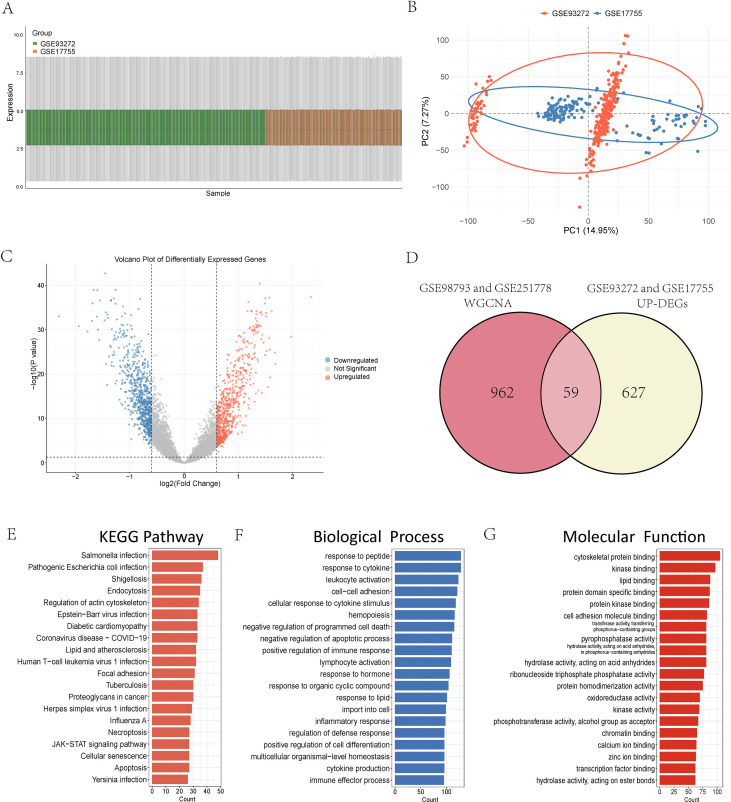
Data preprocessing and identification of candidate comorbidity genes. **(A)** Box plot displaying the standardized expression data across RA datasets. **(B)** PCA results before and after removing batch effects between GSE93272 and GSE17755. **(C)** Volcano plot showing DEGs in the RA combined cohort. **(D)** Venn diagram showing the 59 genes identified from the intersection of MDD lipid-related module genes and RA-upregulated DEGs. **(E)** KEGG pathway analysis. **(F, G)** GO enrichment analysis.

To identify potential markers associated with RA progression, the RA cohorts GSE93272 and GSE17755 were included, normalized, and merged for downstream analyses (**Figure 5A**). Principal component analysis (PCA) indicated distinct batch effects between the GSE93272 and GSE17755 cohorts prior to integration. Following the implementation of the ComBat algorithm, post-correction PCA demonstrated successful data alignment and batch-effect removal, ensuring the integrity of the combined dataset for identifying candidate comorbidity genes (**Figure 5B**).

Differential expression analysis between RA patients and healthy controls was conducted using the limma R package, with thresholds set at P < 0.05 and |log2 fold change| > 0.6 (**Figure 5C**). A total of 1,434 differentially expressed genes (DEGs) were identified, comprising 686 upregulated and 748 downregulated genes. The 686 upregulated DEGs (RA-DEGs) were further subjected to pathway enrichment analysis (**Figures 5E–G**).

Focusing on upregulated RA-DEGs and intersecting them with the MDD lipid metabolism–related gene set, 59 candidate comorbidity-related genes were obtained for subsequent analyses (**Figure 5D**).

### 3.4 Model interpretation and network topology of the signature genes

**Figure 6:**
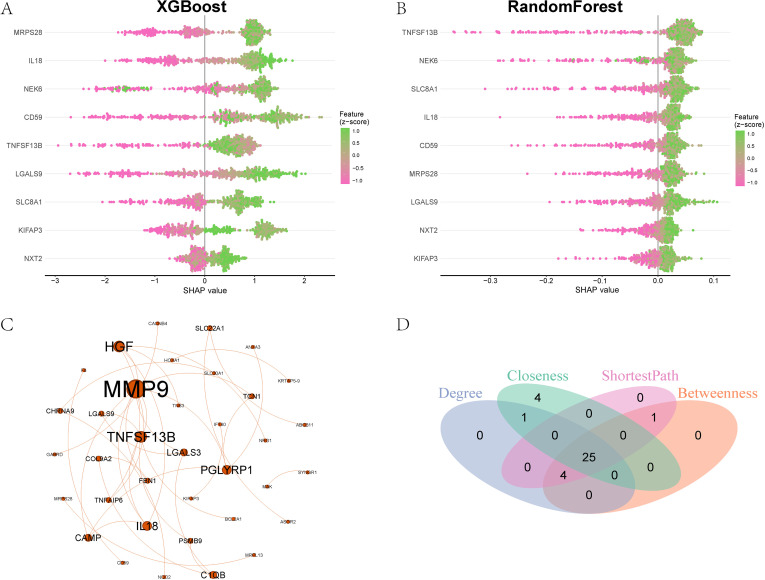
Identification of comorbidity-related hub genes. **(A, B)** SHAP summary plots for the top 10 predictors in the XGBoost **(A)** and random forest **(B)** models; the x-axis shows SHAP values and colors indicate relative gene expression. **(C)** PPI network of the 59 shared candidate genes. **(D)** Venn diagram of hub genes selected by four topological indices (degree, closeness, betweenness, and shortest path); the intersecting genes are defined as core comorbidity-related hub genes.

To understand the contribution of each gene to RA classification, we first refined the 59 candidate genes to a core set of nine feature genes using LASSO regression (see Methods). Using this nine-gene signature, we constructed XGBoost and Random Forest models. SHapley Additive exPlanations (SHAP) analysis, based on the XGBoost model, visualized the magnitude and direction of each gene’s impact on the prediction (**Figures 6A, B**). Among them, IL18, TNFSF13B, and CD59 consistently showed high importance scores in both models.

To explore the functional relationships among the broader candidate set, we constructed a protein-protein interaction (PPI) network from the initial 59 shared genes. This network revealed a tightly interconnected functional module (**Figure 6C**). Subsequent topological analysis using four centrality measures (Degree, Closeness, Betweenness, and Shortest Path) identified a set of high-confidence hub genes. The intersection of these measures highlighted a core group of genes central to the network (**Figure 6D**). Notably, among the top-ranked hub genes were TNFSF13B, IL18, and CD59, reinforcing their potential as key regulatory nodes within the comorbidity-related molecular network.

### 3.5 Selection and expression validation of the nine-gene signature

Based on the 59 candidate genes, LASSO regression refined the feature set to nine genes most strongly associated with RA: ANXA3, IL18, CD59, TNFSF13B, BMX, WASF1, SLC8A1, COMMD8, and NXT2.

**Figure 7:**
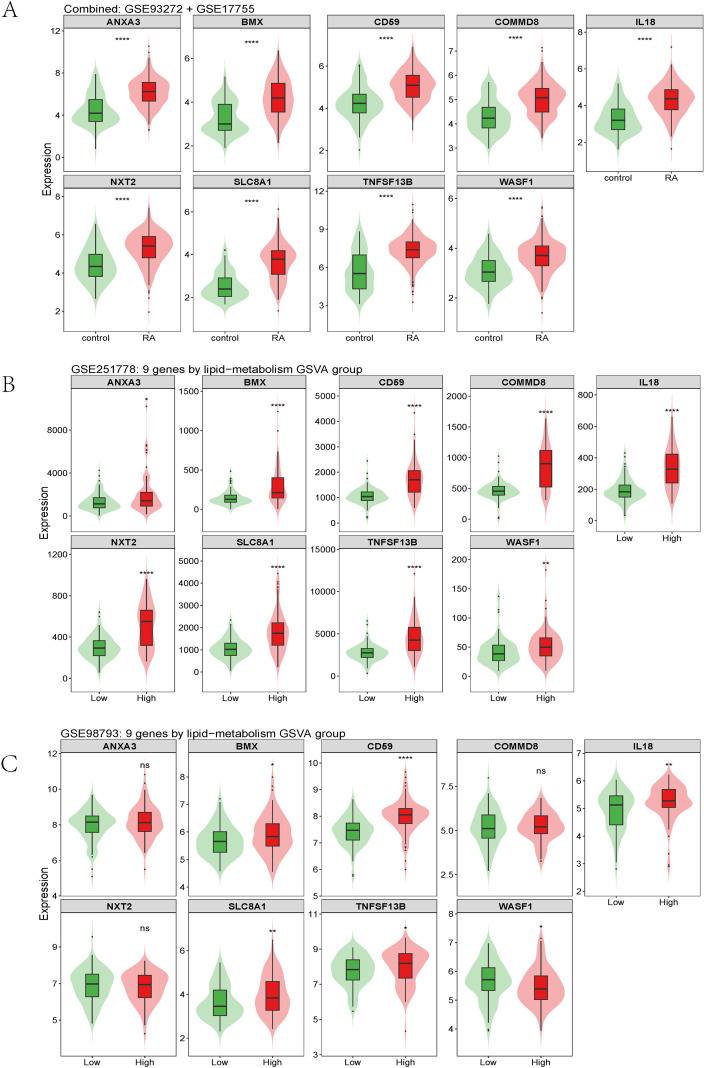
Expression validation of comorbidity-related genes. **(A–C)** Expression levels of core hub genes across RA and MDD cohorts. Statistical significance is indicated as follows: *P < 0.05, **P < 0.01, ****P < 0.0001; ns, not significant.

We first validated the expression patterns of these nine genes. In the RA cohorts, all nine genes were significantly upregulated in patients compared to healthy controls (**Figure 7A**). In the MDD cohorts, their expression levels showed distinct patterns when compared between the high and low lipid metabolism score groups, aligning with the underlying lipid metabolic activity (**Figures 7B, C**). This step confirms the differential expression of the signature genes in both disease contexts.

### 3.6 Predictive performance evaluation of the nine-gene signature

**Figure 8:**
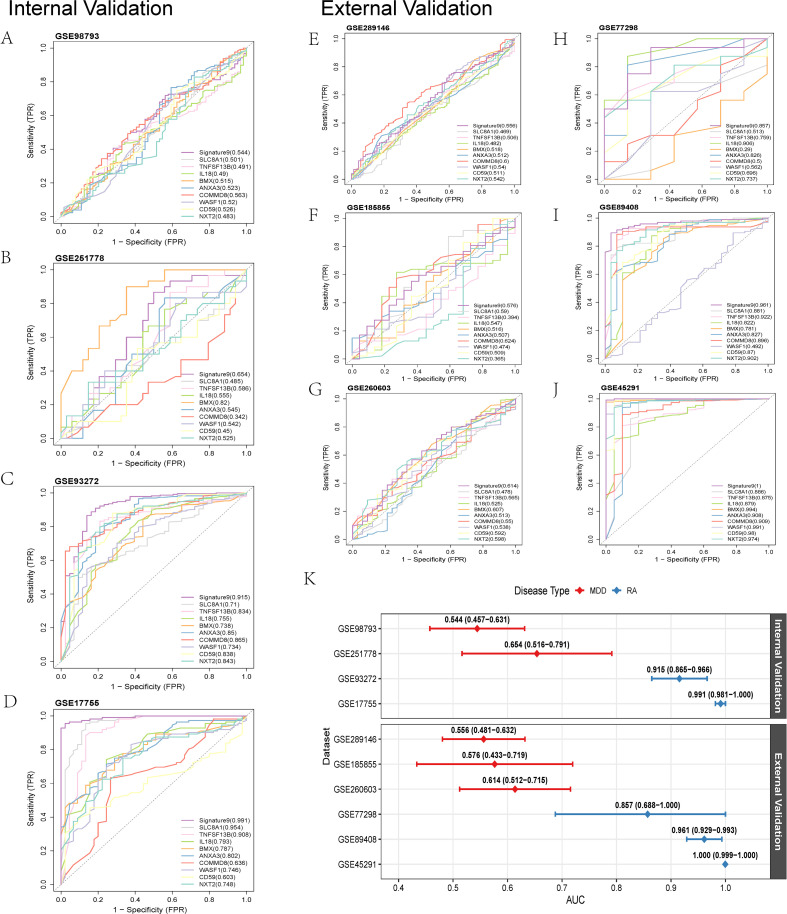
Internal and external validation of the nine-gene XGBoost signature. **(A–D)** Five-fold cross-validation ROC curves of the integrated nine-gene signature in the discovery cohorts (GSE17755, GSE93272, GSE251778, and GSE98793). **(E–J)** ROC curves of the nine-gene signature in six independent external validation cohorts. **(K)** Forest plot summarizing AUCs and 95% confidence intervals of the signature across all datasets, stratified by disease type (MDD vs. RA).

We evaluated the predictive power of the nine-gene signature using XGBoost models. In internal validation with five-fold cross-validation, the signature showed markedly different performance between diseases: it achieved high AUCs in RA discovery cohorts (GSE17755: AUC = 0.991; GSE93272: AUC = 0.915), but only modest discriminative ability in MDD discovery cohorts (GSE98793: AUC = 0.544; GSE251778: AUC = 0.654) (**Figures 8A–D**).

This pattern was further confirmed in independent external validation cohorts. The signature maintained robust performance in RA datasets (**Figures 8H–J**; GSE77298, GSE89408, GSE45291; AUC 0.86–1.00), while its discriminative power remained consistently weak in MDD cohorts (**Figures 8E–G**; GSE289146, GSE185855, GSE260603; AUC 0.55–0.61). A forest-plot summary across all datasets (**Figure 8K**) clearly illustrated this disease-specific performance: the pooled AUC estimate was significantly higher in RA cohorts than in MDD cohorts (RA: AUC = 0.92, 95% CI 0.88–0.96; MDD: AUC = 0.58, 95% CI 0.54–0.62). The remarkable stability of this RA pooled estimate across independent datasets demonstrates that the nine-gene signature maintains high discriminative power despite the technical and population heterogeneity inherent in diverse GEO series.

Collectively, the “strong in RA, weak in MDD” performance profile suggests that this nine-gene signature does not represent a general MDD biomarker.

The discrepancy in predictive accuracy—reflected by a pooled AUC of 0.92 in RA versus 0.58 in MDD—reflects the molecular specificity of the nine-gene signature. This pattern suggests that while the genes were derived from MDD-associated lipid modules, they represent an immune–inflammatory state that is preferentially activated in RA. This reinforces the signature’s role as a potential molecular link to increased comorbidity risk in a specific subset of MDD individuals rather than a general diagnostic tool for MDD. By selectively capturing this immune-activated molecular state that overlaps with core RA pathology, the signature provides a plausible bridge between lipid metabolic dysregulation in psychiatric conditions and autoimmune vulnerability.

### 3.7 Independence of the lipid–immune signature from phenotypic BMI

To determine whether obesity-driven meta-inflammation confounded our molecular findings, we systematically reviewed clinical metadata across all ten cohorts. BMI data were available for four of the five MDD cohorts but were largely unavailable for the RA cohorts. In the MDD cohorts, statistical analysis revealed no significant differences in BMI between MDD patients and healthy controls after *FDR* correction. Furthermore, BMI showed no consistent or significant correlation with the lipid metabolism score, the nine-gene signature score, or the core hub genes *(TNFSF13B*, *IL18*, and *CD59*) across all available datasets (all *FDR* > 0.05; **Supplementary Table 1**). Notably, in the GSE98793 cohort, our signature remained robustly associated with disease status despite BMI being used as a covariate in the original study’s models. These results, together with our *in vitro* findings using the HT-22 cell model (which is devoid of systemic metabolic confounders), collectively support the notion that the identified lipid–immune axis is independent of phenotypic obesity.

### 3.8 Immune infiltration characteristics of the nine feature genes

**Figure 9:**
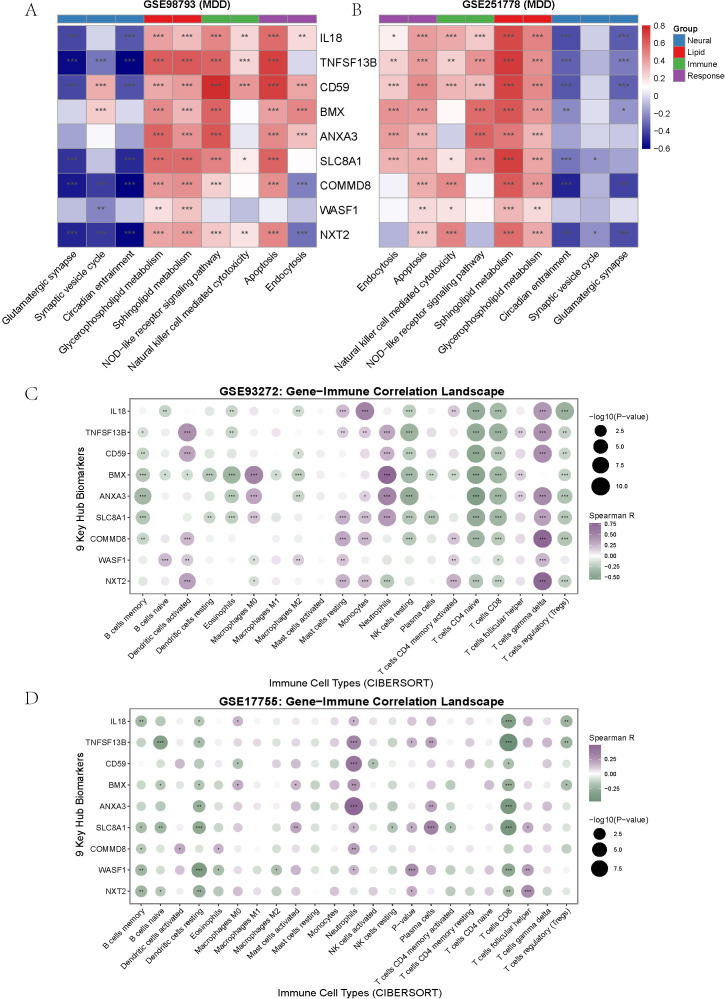
Immune infiltration and pathway correlation analyses. **(A, B)** Heatmaps showing correlations between the nine feature genes and selected metabolic/immune pathways in MDD cohorts GSE98793 **(A)** and GSE251778 **(B)**. **(C, D)** Bubble plots illustrating correlations between the nine feature genes and immune cell infiltration estimated using CIBERSORT in RA cohorts GSE93272 (C) and GSE17755 (D). Statistical significance is indicated as follows: *P < 0.05, **P < 0.01, ***P < 0.001.

In the MDD cohorts GSE98793 and GSE251778, correlations between the nine feature genes and multiple functional pathways were analyzed (**Figures 9A, B**). The results showed that these genes were significantly associated with lipid metabolism–related pathways (e.g., glycerophospholipid and sphingolipid metabolism), immune-related pathways (e.g., NOD-like receptor signaling, NK cell–mediated cytotoxicity), and stress-response pathways. Similar correlation patterns were observed across the two independent cohorts, suggesting a consistent functional association of the nine-gene signature.

In the RA cohorts GSE93272 and GSE17755, correlations between the nine feature genes and immune cell infiltration levels were further evaluated (**Figures 9C, D**). Several genes exhibited significant associations with multiple immune cell types, including monocytes, neutrophils, B cells, and different T cell subsets. Importantly, the correlation patterns were largely consistent between the two RA cohorts, suggesting a robust relationship between the nine-gene signature and the immune microenvironment in RA.

Collectively, these findings indicate that the nine feature genes are not only linked to lipid metabolic pathways but also closely associated with immune cell infiltration, supporting their potential involvement in immune–inflammatory regulation.

### 3.9 Conditioned medium from lipid-stressed neurons activates synovial fibroblasts and upregulates hub genes

Establishment of the HT-22 stress/lipotoxicity model and effects of conditioned medium on FLS.

**Figure 10:**
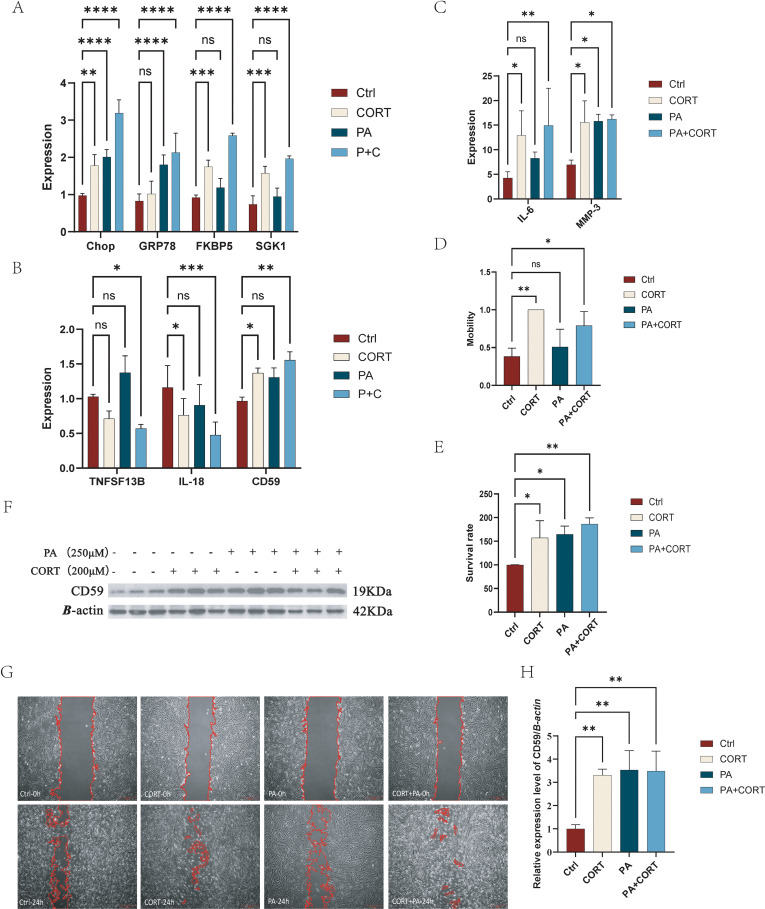
*In vitro* validation of the neuron–synovium axis. **(A)** qPCR analysis of stress-responsive genes in HT-22 cells, validating the stress/lipotoxicity model. **(B)** qPCR analysis of core hub genes (*IL18, TNFSF13B, CD59*) in FLS following CM treatment. **(C)** ELISA-based quantification of IL-6 and MMP3 secretion in FLS culture supernatants. **(D)** Quantification of FLS migration capacity assessed by scratch wound healing assay. **(E)** Cell viability of FLS evaluated by CCK-8 assay. **(F)** Representative Western blot bands showing CD59 protein expression in FLS. **(G)** Representative images of scratch wound healing assays showing wound closure at 0 h and 24 h. **(H)** Densitometric quantification of CD59 protein expression normalized to β-actin. Statistical significance was determined by one-way ANOVA. *P < 0.05, **P < 0.01, ***P < 0.001, ****P < 0.0001; ns, not significant.

As shown in **Figure 10A**, the expression levels of stress-related genes in HT-22 cells were significantly upregulated in the treatment groups compared to the control, supporting the establishment of a neuronal stress model. Specifically, cells treated with corticosterone (CORT) exhibited elevated expression of glucocorticoid-responsive genes (FKBP5, SGK1), validating the induction of glucocorticoid receptor pathway activation. Cells exposed to palmitic acid (PA) showed increased expression of endoplasmic reticulum stress markers (CHOP, GRP78), confirming the induction of lipotoxic stress. Critically, the combined treatment (P+C) led to a synergistic upregulation of all four stress markers, suggesting that CORT and PA together are associated with a composite cellular stress state characterized by concurrent glucocorticoid signaling and lipotoxic/ER stress responses. These results suggest that the combined treatment provides an experimental *in vitro* framework to explore the integrated stress and lipid metabolic dysregulation associated with MDD.

At the FLS level, RT-qPCR analysis demonstrated that stimulation with HT-22-derived conditioned medium modulated the mRNA expression of all three hub genes: *TNFSF13B*, *IL18*, and *CD59*. Among these, *CD59* exhibited the most consistent and significant upregulation, particularly in the P+C-CM group. In contrast, *IL18* and *TNFSF13B* showed treatment-dependent downregulation, suggesting that while these genes are central to the overall comorbidity signature, their induction in synoviocytes may be limited by cell-type-specific regulatory programs.

Functional analyses of FLS are shown in **Figures 10C–E**. ELISA results demonstrated that, compared with the control group, both CORT-CM and PA-CM increased the secretion of the inflammatory cytokine IL-6 and the matrix-degrading protease MMP-3. The highest levels of IL-6 and MMP-3 were observed in the P+C-CM–treated group (**Figure 10C**). Quantitative analysis of scratch assays showed that CM treatment significantly affected FLS migratory capacity, with the P+C-CM group exhibiting the highest migration rate (**Figure 10D**). In addition, CCK-8 assays revealed that different CM treatments altered FLS cell viability, and the highest viability was observed in the P+C-CM group (**Figure 10E**). Together, these results indicate that HT-22-derived CM is associated with altered inflammatory and migratory properties of FLS.

Following the transcriptional analysis, CD59 protein expression in FLS was further examined by Western blotting. As shown in **Figure 10F**, CD59 protein was detectable in all groups, with visibly stronger band intensity observed in FLS treated with CM derived from CORT-, PA-, and P+C-treated HT-22 cells compared with the control group. Among these groups, CM from the P+C-treated HT-22 cells produced the most prominent increase in CD59 protein expression.

Representative images of the scratch wound healing assay are shown in **Figure 10G**. At 0 h, scratch widths were comparable among all groups. At 24 h, clear differences in wound closure were observed, with the P+C-CM group showing the most pronounced scratch closure, consistent with the quantitative analysis.

Densitometric analysis of the Western blot results further confirmed these observations (**Figure 10H**). Quantification of CD59 protein levels, normalized to β-actin, demonstrated a significant increase in CD59 expression in all CM-treated groups relative to the control group. Consistent with the mRNA expression pattern, the highest CD59 protein level was observed in the P+C-CM group. These results indicate that CM derived from stress- and lipotoxicity-exposed HT-22 cells promotes upregulation of CD59 at both the transcriptional and protein levels in FLS.

## 4 Discussion

### 4.1 Main findings

This study established a cross-disease integrative framework to identify lipid-immune molecular features linking MDD-associated lipid metabolism with RA comorbidity risk. Using multi-cohort transcriptomic data, we systematically identified and validated these features through the following key steps:

First, in MDD cohorts, we identified co-expression modules strongly associated with lipid metabolism by integrating single-sample gene set enrichment analysis (ssGSEA) with weighted gene co-expression network analysis (WGCNA). Intersecting these modules with differentially expressed genes from merged RA cohorts yielded 59 candidate genes. Subsequent refinement via LASSO regression produced a nine-gene signature (ANXA3, IL18, CD59, TNFSF13B, BMX, WASF1, SLC8A1, COMMD8, NXT2), which demonstrated stable discriminative power in RA cohorts. This signature was further validated and visualized using machine learning methods, including random forest and XGBoost. Notably, this multi-stage integrated screening framework was specifically designed to transcend simple correlative observations. By systematically reducing biological noise through GeneCards filtering and WGCNA module locking, followed by statistical regularization via LASSO and machine learning, we ensured that the final nine-gene signature was not determined by a single arbitrary cutoff but by a reproducible and parsimonious selection process. Such a robust approach confirms that the core hub genes, specifically *TNFSF13B*, *IL18*, and *CD59*, represent highly stable regulatory nodes at the lipid–immune interface.

Protein-protein interaction (PPI) network and topological analyses identified 25 high-centrality hub genes. Among these, TNFSF13B, IL18, and CD59 were consistently highlighted across differential expression, machine learning selection, and network topology analyses, indicating their role as high-confidence core nodes. Notably, the nine-gene signature exhibited high predictive accuracy (AUC) in RA cohorts but only weak, near-random discriminative ability across MDD cohorts. This “strong in RA, weak in MDD” pattern suggests that the signature is not a general MDD biomarker. Instead, it likely reflects an immune-activation–related molecular state that overlaps with core RA pathology and is present only in a subset of MDD individuals, potentially contributing to their increased comorbidity risk.

### 4.2 Comparison with previous studies and study extension

These findings must be contextualized within the existing literature. Previous epidemiological and inflammatory studies have established a bidirectional link between MDD and RA, implicating shared disturbances in inflammatory cytokine networks and lipid metabolism. However, most prior work has focused on single diseases or isolated pathways, lacking integrative molecular evidence that consistently connects both conditions across independent cohorts.

In contrast, our study extends beyond simple cross-disease comparisons. We adopted a sequential, hypothesis-driven approach: starting from lipid metabolism–related co-expression modules in MDD, cross-validating findings in multi-cohort RA data, and further refining results through machine learning and network topology analyses to identify a stable molecular signature. This strategy systematically integrates lipid dysregulation, immune activation, and RA risk within a unified analytical framework, providing molecular-level support for a testable lipid–immune–driven comorbidity axis.

### 4.3 Mechanistic hypothesis

A Lipid–Immune Coupling Framework in RA–MDD Comorbidity.

Based on our integrative multi-omics analyses, we propose a hierarchical mechanistic hypothesis centered on three lipid–immune hub genes: *TNFSF13B*, *IL18*, and *CD59.* While our transcriptomic data show consistent cross-disease associations, the specific molecular mechanisms should be interpreted as biologically plausible and supported by existing literature. Specifically, we propose a “membrane-to-nucleus” integration model in which altered cholesterol and sphingolipid content—a hallmark of lipid stress in MDD—facilitates the clustering of TNFSF13B (BAFF) receptors and the anchoring of CD59 within lipid rafts (membrane microdomains). This structural shift may directly modulate immune signaling and complement inhibition in the synovium, providing a testable framework for psychiatric–immune comorbidity. This framework is organized into three functionally interlinked axes:

1. Lipid-Sensitive Inflammatory Amplification Axis (IL18, TNFSF13B).
  This axis links lipid metabolic stress to enhanced inflammatory and humoral immune signaling. IL-18, a key pro-inflammatory cytokine, is regulated by sphingolipid metabolism (e.g., ceramide-driven inflammasome activation) and forms a feed-forward loop that amplifies systemic inflammation in both metabolic and autoimmune contexts (30–34). TNFSF13B (BAFF), essential for B-cell survival and autoantibody production, signals through cholesterol- and sphingolipid-enriched membrane microdomains (lipid rafts). Altered lipid composition may therefore facilitate BAFF receptor clustering and potentiate B-cell activation, linking lipid dysregulation to humoral autoimmunity (35–37).
2. Membrane Microenvironment & Immune Homeostasis Axis (CD59, ANXA3).
  Downstream of inflammatory triggers, lipid changes directly affect membrane architecture and its associated regulators. CD59, a GPI-anchored inhibitor of the membrane attack complex (MAC), is localized in lipid rafts and is functionally sensitive to membrane cholesterol and sphingolipid content; its dysregulation can lead to unchecked complement activity and tissue injury (38–42). ANXA3, a Ca^2+^-dependent phospholipid-binding protein, contributes to membrane stabilization, repair, and inflammatory signal modulation. Its expression changes under stress may reflect an adaptive or maladaptive response to lipid-altered membrane integrity and inflammation (43–45). Together, they represent a critical interface where lipid metabolism intersects with membrane-localized immune checkpoint and damage control.
3. Intracellular Signal Integration Network (BMX, WASF1, SLC8A1, COMMD8, NXT2).
  These genes represent adaptive and regulatory responses to lipid–immune dysregulated signaling. They are implicated in PI3K/Akt signaling (BMX), actin cytoskeleton remodeling (WASF1), ion homeostasis (SLC8A1), NF-κB feedback (COMMD8), and nucleocytoplasmic transport (NXT2), collectively forming an intracellular network that may sustain and integrate inflammatory outputs under lipid stress (46–52).

Focus on Triple-Validated Hub Genes.

Among the nine signature genes, TNFSF13B, IL18, and CD59 were consistently identified across differential expression, machine learning, and network topological analyses. They respectively represent humoral immune amplification, classical inflammatory drive, and complement checkpoint regulation—all mechanistically coupled to lipid microenvironmental changes. We therefore propose them as central “lipid–immune hubs” in RA–MDD comorbidity (30, 39, 53–56).

We propose this putative lipid–immune coupling framework to explain the molecular interface between MDD and RA. While the consistency across independent cohorts and our functional validation in FLS point toward a shared pathway, these results should be interpreted as a mechanistic hypothesis rather than established causality. The observed upregulation of hub genes such as *IL18* and *TNFSF13B* indicates a molecular state that may bridge psychiatric stress and synovial pathology, but the precise causal hierarchy remains to be fully determined. Consequently, while these core hub genes nominate a potential mechanistic entry point linking lipid dysregulation in MDD with immune-inflammatory pathology in RA, it is important to note that this hypothesis is derived primarily from transcriptomic associations; thus, the specific molecular mechanisms and causal relationships await further large-scale longitudinal and experimental validation.

Complementing these findings, our immune infiltration analysis provides preliminary cellular-level insights aligning with the proposed framework. In two independent RA cohorts, the expression levels of all nine signature genes correlated significantly with the infiltration abundance of monocytes, neutrophils, B cells, and several T-cell subsets. These correlations suggest that these genes may be involved in immune cell recruitment, activation, or functional modulation. Furthermore, pathway correlation analyses revealed that these genes are closely linked with both lipid metabolism-related pathways and immune-inflammatory signaling pathways. Together, these results support the hypothesis that these signature genes participate in a putative pathogenic cascade of “lipid metabolic dysregulation → immune microenvironment remodeling → inflammatory amplification.” By connecting gene expression patterns with shifts in immune cell composition, our infiltration analysis provides more direct and systematic biological evidence for the lipid–immune coupling mechanism proposed herein, advancing our understanding of how lipid abnormalities in MDD may be linked to RA immune pathology.

### 4.4 Bidirectional feedback: the synovium-to-neuron conduit

Although the present study focuses on the neuron-to-synovium direction, the MDD-RA relationship is now widely recognized as a dynamic, bidirectional axis rather than a unidirectional cascade. A reverse synovium-to-neuron pathway (a “bottom-up” trajectory) may contribute to RA-associated depressive symptoms and the perpetuation of MDD-RA comorbidity. In the inflamed synovium, activated FLS and immune cells release a potent secretome comprising cytokines such as TNF-α, IL-6, and IL-1β that can enter the systemic circulation, signal across the blood-brain barrier, and drive neuroinflammation. Recent evidence has highlighted that distinct fibroblast subsets drive tissue damage (57) and that synovial fibroblast gene expression is directly associated with sensory nerve growth and inflammatory pain in RA (58), providing a molecular foundation for joint-to-brain communication.

Altered lipid metabolism may provide a critical regulatory layer in this reverse communication. Both MDD and RA exhibit convergent lipidomic signatures involving lysophosphatidylcholine (LPC), phosphatidylcholine (PC), and sphingomyelins (SM). This “lipid paradox,” whereby chronic inflammation reshapes systemic lipid profiles (59, 60), may further disrupt central nervous system (CNS) homeostasis by altering neuronal membrane fluidity and synaptic vesicle trafficking (61).

Beyond the joint–brain axis, the systemic lipid–immune dysregulation identified here may contribute to the accelerated atherosclerosis observed in RA. Clinical data from the ESCAPE RA study and the CANTOS trial underscore that inflammation and psychiatric distress can drive CV risk independently of conventional lipid measures (62, 63). Notably, several hub genes in our signature are central to vascular pathology: *IL18* is a recognized driver of atherosclerotic plaque instability (64, 65), while *CD59* provides indispensable protection to the vascular endothelium against complement-mediated damage (66). Furthermore, the role of *TNFSF13B* (BAFF) signaling in promoting atherosclerosis highlights a potential link between systemic B-cell activation and vascular events. These findings imply that the lipid-immune-neural axis may serve as a molecular bridge linking neuro-metabolic stress to the heightened cardiovascular mortality in RA. Direct experimental validation of this “bottom-up” feedback remains an important priority for future translational research.

### 4.5 Lipid-modulated inflammatory execution in synoviocytes

To experimentally test the proposed ‘neuron-to-synovium’ axis within the lipid–immune framework identified from transcriptomic analyses of RA–MDD comorbidity, we performed the following *in vitro* functional assays. A combined glucocorticoid and saturated fatty acid stimulation was used to establish a stress–lipotoxicity model in HT-22 hippocampal neurons, generating a stable stress-altered neuronal state.

Conditioned medium from stressed HT-22 cells induced reproducible molecular and functional changes in fibroblast-like synoviocytes. These results indicate that neuronal stress accompanied by lipid dysregulation can modulate synovial inflammatory phenotypes through paracrine signaling. Although the specific mediators were not identified, the findings support the presence of a neuron–synovium communication axis.

The partial discordance in the expression of the three hub genes in our FLS model reinforces the importance of cell-type specificity. While *CD59* was consistently upregulated, *IL18* and *TNFSF13B* were downregulated in this specific cell type. This is biologically plausible as *IL-18* and *TNFSF13B* (*BAFF*) are fundamentally associated with myeloid and B-cell inflammatory activity, respectively, and FLS are not their primary cellular source in the rheumatoid joint (67, 68). Importantly, the robust induction of classical inflammatory effectors such as IL-6 and MMP-3, combined with the validated upregulation of *CD59* at both the transcriptional and protein levels, confirms that neuronal stress-related signals are sufficient to activate synovial inflammatory programs.

Notably, CD59 upregulation was confirmed at both the mRNA and protein levels, with Western blot analysis demonstrating increased CD59 protein expression in FLS. As an inhibitor of complement membrane attack complex formation, elevated CD59 suggests remodeling of membrane-associated immune regulation under lipid- and stress-related conditions.

Importantly, while our functional experiments demonstrate that neuronal stress alters FLS inflammatory phenotypes via paracrine signaling, the identity of the mediators (e.g., specific lipid species or extracellular vesicles) responsible for this effect has not been identified in the present study. The observed activation of synovial inflammatory programs (IL-6, MMP-3) and the upregulation of CD59 provide functional validation of the axis, but the receptor-level mechanisms underlying this effect and the causal roles of IL18 and TNFSF13B in synoviocytes remain speculative.

Together with enhanced migration and altered cell viability, these findings suggest that lipid dysregulation does not uniformly activate all comorbidity-associated inflammatory genes in FLS. Instead, it selectively promotes inflammatory and invasive effector phenotypes. This selective immune remodeling provides functional support for lipid-associated pathways linking stress-related psychiatric conditions with RA synovial pathology.

### 4.6 Therapeutic potential and clinical implications

The discovery of the lipid–immune–neural axis provides a basis for precision stratification of MDD patients, identifying a high-risk phenotype with increased susceptibility to autoimmune transition. Specifically, core hub genes such as *IL18* and *TNFSF13B* represent potential nodes for intercepting the paracrine loop between the CNS and the joint environment. Although these findings are hypothesis-generating, they suggest that future translational research should evaluate whether targeting these lipid–immune pathways—or suppressing “lipid–stress” signals through metabolic and lifestyle interventions—may provide a novel pharmacological or preventive approach for high-risk psychiatric populations.

### 4.7 Limitations and future perspectives

While this study provides a novel, multi-level framework linking lipid dysregulation in MDD to immune-inflammatory pathology in RA, several limitations should be acknowledged to contextualize the findings and guide future work. First, the bioinformatic insights are derived from heterogeneous public cohorts, which may limit generalizability. A significant constraint is the inherent heterogeneity and residual confounding associated with public transcriptomic datasets; specifically, included cohorts differed in platforms, sample processing, and clinical characteristics. Furthermore, key clinical covariates—including BMI, smoking status, metabolic comorbidities, and medication history (e.g., antidepressants, glucocorticoids, and DMARDs)—were not consistently available and could not be uniformly adjusted for. These factors may have influenced the expression of lipid-related and immune-inflammatory genes, such as *IL18* and *TNFSF13B*. Related to this data heterogeneity, the observed discriminative performance across general MDD cohorts warrants careful interpretation in light of the study design. The signature was not developed as a general MDD diagnostic classifier, but as an RA-oriented lipid–immune signature derived from MDD lipid-related modules. Given that MDD is a highly heterogeneous condition, our findings support the interpretation that this signature identifies an “RA-like” comorbidity-risk phenotype selectively present in a specific subset of individuals. These individuals may represent a high-risk group where lipid-mediated inflammatory reprogramming, characterized by core hubs like CD59, IL6, and MMP3, provides a testable mechanistic conduit for psychiatric–immune comorbidity. Second, the cross-sectional nature of the transcriptomic data precludes causal inference for the proposed “MDD–lipid–immune–RA” pathway. It is important to acknowledge that the transcriptomic signatures identified here represent associative patterns within the current datasets. Given the complex bidirectional nature of the brain-joint axis, we cannot exclude the possibility that systemic inflammation also feeds back into lipid metabolic disturbances. Future research utilizing longitudinal clinical cohorts or Mendelian Randomization analysis will be essential to disentangle the causal directions within this lipid–immune network. Third, while our molecular stratification effectively captured transcriptional lipid activity across multiple cohorts, we acknowledge the potential influence of obesity-related low-grade inflammation. Although our supplemental analyses suggested that the identified signature is not primarily driven by BMI in the MDD cohorts with available data, the lack of granular BMI data in the RA cohorts remains a limitation. Fourth, it must be emphasized that the present study was not designed as a therapeutic or preventive intervention trial. We did not include pharmacological treatments or longitudinal follow-up for RA development. Therefore, our current data do not allow for established clinical recommendations. Future prospective studies are required to validate whether metabolic optimization aimed at reducing lipid stress can effectively prevent the transition from MDD to overt systemic autoimmunity. Finally, although our *in vitro* model functionally validates a neuron-to-synovium paracrine axis, the *in vivo* relevance and the specific lipid-altered mediators within the conditioned medium remain to be identified. Specifically, while *IL18* and *TNFSF13B* were experimentally assessed at the mRNA level, their protein-level and functional roles in FLS were not fully confirmed in the current simplified model. Future investigations utilizing multi-cellular systems, such as macrophage-FLS or B-cell-FLS co-culture models, will be essential to capture the full paracrine activation and functional coordination of the lipid-immune interface in RA-MDD comorbidity. Ultimately, although our data support a lipid–immune neuro–synovial communication axis, several mechanistic steps remain speculative. In particular, the causal role of specific membrane microdomains and the precise identity of lipid–altered paracrine mediators in neuronal conditioned medium have not been directly tested. Future investigations using lipidomics, mediator-blocking experiments, and *in vivo* models are essential to transform these hypothesis-driven claims into established mechanistic evidence.

Future *in vivo* validation is essential to interrogate the proposed lipid–immune–neuro–synovial axis under pathophysiologically relevant conditions. One promising strategy is to integrate MDD-like paradigms, including chronic unpredictable mild stress (CUMS) or chronic glucocorticoid exposure, with established experimental arthritis models such as collagen-induced arthritis (CIA) (69, 70). These combinatorial models would enable systematic evaluation of whether stress- or lipid-driven metabolic perturbations exacerbate joint inflammation and immune cell infiltration. Disease progression should be assessed using standardized clinical arthritis scores, detailed synovial histopathology, and quantification of key inflammatory mediators, including IL-6 and MMP-3. Given the well-established contribution of neuro–immune interactions to rheumatoid arthritis pain and its overlap with affective disorders, it will also be critical to incorporate neural readouts. In particular, activation of dorsal root ganglia (DRG) neurons and the expression of nociceptive markers such as calcitonin gene-related peptide (CGRP) and transient receptor potential vanilloid 1 (TRPV1) should be monitored to validate bidirectional communication between the peripheral joint environment and the nervous system (71). To establish causality, interventional approaches are required. These may include tissue- or cell-type-specific perturbation of hub genes—such as *TNFSF13B* (*BAFF*), *IL18*, and *CD59*—using adeno-associated viral (AAV) vectors or conditional genetic models. In parallel, pharmacological blockade of *BAFF* or *IL-18* signaling pathways should be evaluated for their efficacy in ameliorating both joint pathology and depression-like behaviors. Such experiments will be crucial to determine whether the identified lipid–immune signature represents a mechanistic driver, rather than a secondary correlate, of MDD–RA comorbidity (53, 72).

These limitations, however, define clear and compelling directions for subsequent research. Building directly on the core discoveries of this work—the conserved lipid–immune signature and the validated neuro–synovial axis—future investigations should prioritize: (i) mechanistic dissection of the identified hub genes (particularly TNFSF13B, IL18, and CD59) using genetic and pharmacological tools in lipid-dysregulated microenvironments *in vivo*; (ii) cellular deconvolution via single-cell or spatial omics to pinpoint the precise tissue and immune cell subsets orchestrating this cross-talk; and (iii) translational validation in prospective cohorts integrating lipidomics and immune profiling to establish the predictive value of the signature and explore early intervention strategies.

In conclusion, by integrating computational discovery with experimental validation, this work integrates computational discovery with preliminary experimental validation to propose a testable, lipid-centric mechanistic hypothesis for MDD–RA comorbidity. It not only nominates novel molecular hubs but also provides initial functional support for a direct neuro–synovial communication axis. The experimentally demonstrated neuron–synovium paracrine axis offers a novel mechanistic framework for cross-disease pathogenesis. However, the molecular identity of these conduits remains to be defined, yet provides a rationale for future translational studies aimed at disrupting the lipid–immune link in MDD–RA comorbidity.

## Data Availability

The datasets presented in this study can be found in online repositories. The names of the repository/repositories and accession number(s) can be found in the article/**Supplementary Material**.

## References

[1] Beurel E Toups M Nemeroff CB . The bidirectional relationship of depression and inflammation: Double trouble. Neuron. (2020) 107:234–56. doi: 10.1016/j.neuron.2020.06.002. PMID: 32553197 PMC7381373

[2] Bernal-Vega S Garcia-Juarez M Camacho-Morales A . Contribution of ceramides metabolism in psychiatric disorders. J Neurochem. (2023) 164:708–24. doi: 10.1111/jnc.15759. PMID: 36630272

[3] Sturgeon JA Finan PH Zautra AJ . Affective disturbance in rheumatoid arthritis: Psychological and disease-related pathways. Nat Rev Rheumatol. (2016) 12:532–42. doi: 10.1038/nrrheum.2016.112. PMID: 27411910 PMC5449457

[4] Scott IC Machin A Mallen CD Hider SL . The extra-articular impacts of rheumatoid arthritis: Moving towards holistic care. BMC Rheumatol. (2018) 2:32. doi: 10.1186/s41927-018-0039-2. PMID: 30886982 PMC6390577

[5] Covic T Cumming SR Pallant JF Manolios N Emery P Conaghan PG . Depression and anxiety in patients with rheumatoid arthritis: Prevalence rates based on a comparison of the Depression, Anxiety and Stress Scale (DASS) and the hospital, Anxiety and Depression Scale (HADS). BMC Psychiatry. (2012) 12:6. doi: 10.1186/1471-244X-12-6. PMID: 22269280 PMC3285517

[6] Lu MC Guo HR Lin MC Livneh H Lai NS Tsai TY . Bidirectional associations between rheumatoid arthritis and depression: A nationwide longitudinal study. Sci Rep. (2016) 6:20647. doi: 10.1038/srep20647. PMID: 26857028 PMC4746638

[7] Vallerand IA Lewinson RT Frolkis AD Lowerison MW Kaplan GG Swain MG . Depression as a risk factor for the development of rheumatoid arthritis: A population-based cohort study. RMD Open. (2018) 4:e000670. doi: 10.1136/rmdopen-2018-000670. PMID: 30018804 PMC6045711

[8] Nerurkar L Siebert S McInnes IB Cavanagh J . Rheumatoid arthritis and depression: An inflammatory perspective. Lancet Psychiatry. (2019) 6:164–73. doi: 10.1016/S2215-0366(18)30255-4. PMID: 30366684

[9] Goldsmith DR Rapaport MH Miller BJ . A meta-analysis of blood cytokine network alterations in psychiatric patients: Comparisons between schizophrenia, bipolar disorder and depression. Mol Psychiatry. (2016) 21:1696–709. doi: 10.1038/mp.2016.3. PMID: 26903267 PMC6056174

[10] Liu Y Ho RCM Mak A . Interleukin (IL)-6, tumour necrosis factor alpha (TNF-α) and soluble interleukin-2 receptors (sIL-2R) are elevated in patients with major depressive disorder: A meta-analysis and meta-regression. J Affect Disord. (2012) 139:230–9. doi: 10.1016/j.jad.2011.08.003. PMID: 21872339

[11] Wang Y Chen H Wang J Chen S Liu J Chen X . Association between abnormal plasma lipid metabolism and psychological characteristics in adolescents with major depressive disorder. Depression Anxiety. (2025) 2025:5564796. doi: 10.1155/da/5564796. PMID: 40390837 PMC12088845

[12] Miao G Deen J Struzeski JB Chen M Zhang Y Cole SA . Plasma lipidomic profile of depressive symptoms: A longitudinal study in a large sample of community-dwelling American Indians in the strong heart study. Mol Psychiatry. (2023) 28:2480–9. doi: 10.1038/s41380-023-01948-w. PMID: 36653676 PMC10753994

[13] Tkachev A Stekolshchikova E Golubova A Serkina A Morozova A Zorkina Y . Screening for depression in the general population through lipid biomarkers. EBioMedicine. (2024) 110:105455. doi: 10.1016/j.ebiom.2024.105455. PMID: 39571307 PMC11617895

[14] Liu X Li J Zheng P Zhao X Zhou C Hu C . Plasma lipidomics reveals potential lipid markers of major depressive disorder. Anal Bioanal Chem. (2016) 408:6497–507. doi: 10.1007/s00216-016-9768-5. PMID: 27457104

[15] Koh JH Yoon SJ Kim M Cho S Lim J Park Y . Lipidome profile predictive of disease evolution and activity in rheumatoid arthritis. Exp Mol Med. (2022) 54:143–55. doi: 10.1038/s12276-022-00725-z. PMID: 35169224 PMC8894401

[16] Li R Koh JH Park WJ Choi Y Kim WU . Serum and urine lipidomic profiles identify biomarkers diagnostic for seropositive and seronegative rheumatoid arthritis. Front Immunol. (2024) 15:1410365. doi: 10.3389/fimmu.2024.1410365. PMID: 38765010 PMC11099275

[17] Wang Y Ma L He J Gu H Zhu H . Identification of cancer stem cell-related genes through single cells and machine learning for predicting prostate cancer prognosis and immunotherapy. Front Immunol. (2024) 15:1464698. doi: 10.3389/fimmu.2024.1464698. PMID: 39267762 PMC11390519

[18] Li Y Maher P Schubert D . A role for 12-lipoxygenase in nerve cell death caused by glutathione depletion. Neuron. (1997) 19:453–63. doi: 10.1016/s0896-6273(00)80953-8. PMID: 9292733

[19] Zeng J Xie Z Chen L Peng X Luan F Hu J . Rosmarinic acid alleviate CORT-induced depressive-like behavior by promoting neurogenesis and regulating BDNF/TrkB/PI3K signaling axis. Biomed Pharmacother = Biomed Pharmacother. (2024) 170:115994. doi: 10.1016/j.biopha.2023.115994. PMID: 38070249

[20] Gao X Sun H Hao S Sun H Ge J . Melatonin protects HT-22 cells against palmitic acid-induced glucolipid metabolic dysfunction and cell injuries: Involved in the regulation of synaptic plasticity and circadian rhythms. Biochem Pharmacol. (2023) 217:115846. doi: 10.1016/j.bcp.2023.115846. PMID: 37804870

[21] Zhang Y Xue R Zhang Z Yang X Shi H . Palmitic and linoleic acids induce ER stress and apoptosis in hepatoma cells. Lipids Health Dis. (2012) 11:1. doi: 10.1186/1476-511X-11-1. PMID: 22221411 PMC3306830

[22] Kim M Lee H Lee C Cho S Um MY . Standardized rice bran supplement ameliorates depressive behaviors via FKBP5 mediated glucocorticoid receptor signaling. NPJ Sci Food. (2025) 9:238. doi: 10.1038/s41538-025-00602-9. PMID: 41253845 PMC12627636

[23] Qin Y Cai ML Jin HZ Huang W Zhu C Bozec A . Age-associated B cells contribute to the pathogenesis of rheumatoid arthritis by inducing activation of fibroblast-like synoviocytes via TNF-α-mediated ERK1/2 and JAK-STAT1 pathways. Ann Rheumatic Dis. (2022) 81:1504–14. doi: 10.1136/ard-2022-222605. PMID: 35760450

[24] Galic S Fullerton MD Schertzer JD Sikkema S Marcinko K Walkley CR . Hematopoietic AMPK β1 reduces mouse adipose tissue macrophage inflammation and insulin resistance in obesity. J Clin Invest. (2011) 121:4903–15. doi: 10.1172/JCI58577. PMID: 22080866 PMC3226000

[25] Wobser H Dorn C Weiss TS Amann T Bollheimer C Büttner R . Lipid accumulation in hepatocytes induces fibrogenic activation of hepatic stellate cells. Cell Res. (2009) 19:996–1005. doi: 10.1038/cr.2009.73. PMID: 19546889

[26] Liang CC Park AY Guan JL . *In vitro* scratch assay: A convenient and inexpensive method for analysis of cell migration *in vitro*. Nat Protoc. (2007) 2:329–33. doi: 10.1038/nprot.2007.30. PMID: 17406593

[27] Fan J Schiemer T Vaska A Jahed V Klavins K . Cell via cell viability assay changes cellular metabolic characteristics by intervening with glycolysis and pentose phosphate pathway. Chem Res Toxicol. (2024) 37:208–11. doi: 10.1021/acs.chemrestox.3c00339. PMID: 38191130 PMC10880084

[28] Zeisel MB Druet VA Wachsmann D Sibilia J . MMP-3 expression and release by rheumatoid arthritis fibroblast-like synoviocytes induced with a bacterial ligand of integrin alpha5beta1. Arthritis Res Ther. (2005) 7:R118–26. doi: 10.1186/ar1462. PMID: 15642131 PMC1064889

[29] Carrion M Juarranz Y Perez-Garcia S Jimeno R Pablos JL Gomariz RP . RNA sensors in human osteoarthritis and rheumatoid arthritis synovial fibroblasts: Immune regulation by vasoactive intestinal peptide. Arthritis Rheumatism. (2011) 63:1626–36. doi: 10.1002/art.30294. PMID: 21337319

[30] Somm E Jornayvaz FR . Interleukin-18 in metabolism: From mice physiology to human diseases. Front Endocrinol. (2022) 13:971745. doi: 10.3389/fendo.2022.971745. PMID: 36313762 PMC9596921

[31] Jiang J Shi Y Cao J Lu Y Sun G Yang J . Role of ASM/Cer/TXNIP signaling module in the NLRP3 inflammasome activation. Lipids Health Dis. (2021) 20:19. doi: 10.1186/s12944-021-01446-4. PMID: 33612104 PMC7897379

[32] Leemans JC Cassel SL Sutterwala FS . Sensing damage by the NLRP3 inflammasome. Immunol Rev. (2011) 243:152–62. doi: 10.1111/j.1600-065X.2011.01043.x. PMID: 21884174 PMC3170135

[33] Matsuo T Hashimoto M Ito I Kubo T Uozumi R Furu M . Interleukin-18 is associated with the presence of interstitial lung disease in rheumatoid arthritis: A cross-sectional study. Scand J Rheumatol. (2019) 48:87–94. doi: 10.1080/03009742.2018.1477989. PMID: 30269670

[34] Du X Zou S Yue Y Fang X Wu Y Wu S . Peripheral interleukin-18 is negatively correlated with abnormal brain activity in patients with depression: A resting-state fMRI study. BMC Psychiatry. (2022) 22:531. doi: 10.1186/s12888-022-04176-8. PMID: 35931995 PMC9354267

[35] Mackay F Schneider P Rennert P Browning JL . BAFF AND APRIL: A tutorial on B cell survival. Annu Rev Immunol. (2003) 21:231–64. doi: 10.1146/annurev.immunol.21.120601.141152. PMID: 12427767

[36] Lim CS Lee J Kim JW Lee JO . Highly ordered clustering of TNFα and BAFF ligand-receptor-intracellular adaptor complexes on a lipid membrane. Nat Commun. (2025) 16:5551. doi: 10.1038/s41467-025-61271-6. PMID: 40593711 PMC12216653

[37] Bosello SL Youinou P Daridon C Tolusso B Bendaoud B Pietrapertosa D . Concentrations of BAFF correlate with autoantibody levels, clinical disease activity, and response to treatment in early rheumatoid arthritis. J Rheumatol. (2008) 35:1256–64. 18528969

[38] Voisin TB Couves EC Tate EW Bubeck D . Dynamics and molecular interactions of GPI-anchored CD59. Toxins. (2023) 15:430. doi: 10.3390/toxins15070430. PMID: 37505699 PMC10467114

[39] Couves EC Gardner S Voisin TB Bickel JK Stansfeld PJ Tate EW . Structural basis for membrane attack complex inhibition by CD59. Nat Commun. (2023) 14:890. doi: 10.1038/s41467-023-36441-z. PMID: 36797260 PMC9935631

[40] Fülöp G Brameshuber M Arnold AM Schütz GJ Sevcsik E . Determination of the membrane environment of CD59 in living cells. Biomolecules. (2018) 8:28. doi: 10.3390/biom8020028. PMID: 29772810 PMC6023084

[41] Holers VM Banda NK . Complement in the initiation and evolution of rheumatoid arthritis. Front Immunol. (2018) 9:1057. doi: 10.3389/fimmu.2018.01057. PMID: 29892280 PMC5985368

[42] Luo X Fang Z Lin L Xu H Huang Q Zhang H . Plasma complement C3 and C3a are increased in major depressive disorder independent of childhood trauma. BMC Psychiatry. (2022) 22:741. doi: 10.1186/s12888-022-04410-3. PMID: 36447174 PMC9706857

[43] Gerke V Gavins FNE Geisow M Grewal T Jaiswal JK Nylandsted J . Annexins-a family of proteins with distinctive tastes for cell signaling and membrane dynamics. Nat Commun. (2024) 15:1574. doi: 10.1038/s41467-024-45954-0. PMID: 38383560 PMC10882027

[44] Yang L Lu P Yang X Li K Qu S . Annexin A3, a calcium-dependent phospholipid-binding protein: Implication in cancer. Front Mol Biosci. (2021) 8:716415. doi: 10.3389/fmolb.2021.716415. PMID: 34355022 PMC8329414

[45] Toufiq M Roelands J Alfaki M Syed Ahamed Kabeer B Saadaoui M Lakshmanan AP . Annexin A3 in sepsis: Novel perspectives from an exploration of public transcriptome data. Immunology. (2020) 161:291–302. doi: 10.1111/imm.13239. PMID: 32682335 PMC7692248

[46] Syn N Wang L Sethi G Thiery JP Goh BC . Exosome-mediated metastasis: From epithelial-mesenchymal transition to escape from immunosurveillance. Trends Pharmacol Sci. (2016) 37:606–17. doi: 10.1016/j.tips.2016.04.006. PMID: 27157716

[47] Vanhaesebroeck B Stephens L Hawkins PT . PI3K signalling: The path to discovery and understanding. Nat Rev Mol Cell Biol. (2012) 13:195–203. doi: 10.1038/nrm3290. PMID: 22358332

[48] Takenawa T Suetsugu S . The WASP-WAVE protein network: Connecting the membrane to the cytoskeleton. Nat Rev Mol Cell Biol. (2007) 8:37–48. doi: 10.1038/nrm2069. PMID: 17183359

[49] Dustin ML Choudhuri K . Signaling and polarized communication across the T cell immunological synapse. Annu Rev Cell Dev Biol. (2016) 32:303–25. doi: 10.1146/annurev-cellbio-100814-125330. PMID: 27501450

[50] Blaustein MP Lederer WJ . Sodium/calcium exchange: its physiological implications. Physiol Rev. (1999) 79:763–854. doi: 10.1152/physrev.1999.79.3.763. PMID: 10390518

[51] Hagihara H Yoshikawa Y Ohga Y Takenaka C Murata K Taniguchi S . Na+/Ca2+ exchange inhibition protects the rat heart from ischemia-reperfusion injury by blocking energy-wasting processes. Am J Physiol Heart Circ Physiol. (2005) 288:H1699–707. doi: 10.1152/ajpheart.01033.2004. PMID: 15626686

[52] Maine GN Burstein E . COMMD proteins and the control of the NF kappa B pathway. Cell Cycle (Georgetown Tex). (2007) 6:672–6. doi: 10.4161/cc.6.6.3989. PMID: 17361106 PMC2910620

[53] Mackay F Browning JL . BAFF: a fundamental survival factor for B cells. Nat Rev Immunol. (2002) 2:465–75. doi: 10.1038/nri844. PMID: 12094221

[54] Simons K Toomre D . Lipid rafts and signal transduction. Nat Rev Mol Cell Biol. (2000) 1:31–9. doi: 10.1038/35036052. PMID: 11413487

[55] Hotamisligil GS . Inflammation and metabolic disorders. Nature. (2006) 444:860–7. doi: 10.1038/nature05485. PMID: 17167474

[56] O'Neill LAJ Pearce EJ . Immunometabolism governs dendritic cell and macrophage function. J Exp Med. (2016) 213:15–23. doi: 10.1084/jem.20151570. PMID: 26694970 PMC4710204

[57] Croft AP Campos J Jansen K Turner JD Marshall J Attar M . Distinct fibroblast subsets drive inflammation and damage in arthritis. Nature. (2019) 570:246–51. doi: 10.1038/s41586-019-1263-7. PMID: 31142839 PMC6690841

[58] Bai Z Bartelo N Aslam M Murphy EA Hale CR Blachere NE . Synovial fibroblast gene expression is associated with sensory nerve growth and pain in rheumatoid arthritis. Sci Transl Med. (2024) 16:eadk3506. doi: 10.1126/scitranslmed.adk3506. PMID: 38598614 PMC11931728

[59] Yan J Yang S Han L Ba X Shen P Lin W . Dyslipidemia in rheumatoid arthritis: the possible mechanisms. Front Immunol. (2023) 14:1254753. doi: 10.3389/fimmu.2023.1254753. PMID: 37954591 PMC10634280

[60] Morris G Berk M Walder K O'Neil A Maes M Puri BK . The lipid paradox in neuroprogressive disorders: Causes and consequences. Neurosci Biobehav Rev. (2021) 128:35–57. doi: 10.1016/j.neubiorev.2021.06.017. PMID: 34118292

[61] Vanherle S Loix M Miron VE Hendriks JJA Bogie JFJ . Lipid metabolism, remodelling and intercellular transfer in the CNS. Nat Rev Neurosci. (2025) 26:214–31. doi: 10.1038/s41583-025-00908-3. PMID: 39972160

[62] Liu YL Szklo M Davidson KW Bathon JM Giles JT . Differential association of psychosocial comorbidities with subclinical atherosclerosis in rheumatoid arthritis. Arthritis Care Res. (2015) 67:1335–44. doi: 10.1002/acr.22635. PMID: 26274015 PMC6058701

[63] Ridker PM Everett BM Thuren T MacFadyen JG Chang WH Ballantyne C . Antiinflammatory therapy with canakinumab for atherosclerotic disease. N Engl J Med. (2017) 377:1119–31. doi: 10.1056/NEJMoa1707914. PMID: 28845751

[64] Mallat Z Corbaz A Scoazec A Graber P Alouani S Esposito B . Interleukin-18/interleukin-18 binding protein signaling modulates atherosclerotic lesion development and stability. Circ Res. (2001) 89:E41–5. doi: 10.1161/hh1901.098735. PMID: 11577031

[65] Libby P . Inflammation in atherosclerosis. Nature. (2002) 420:868–74. doi: 10.1038/nature01323. PMID: 12490960

[66] Wu G Hu W Shahsafaei A Song W Dobarro M Sukhova GK . Complement regulator CD59 protects against atherosclerosis by restricting the formation of complement membrane attack complex. Circ Res. (2009) 104:550–8. doi: 10.1161/CIRCRESAHA.108.191361. PMID: 19131645 PMC4267695

[67] Kalled SL . The role of BAFF in immune function and implications for autoimmunity. Immunol Rev. (2005) 204:43–54. doi: 10.1111/j.0105-2896.2005.00219.x. PMID: 15790349

[68] Liew FY McInnes IB . Role of interleukin 15 and interleukin 18 in inflammatory response. Ann Rheumatic Dis. (2002) 61:ii100–2. doi: 10.1136/ard.61.suppl_2.ii100. PMID: 12379638 PMC1766710

[69] Willner P . Chronic mild stress (CMS) revisited: consistency and behavioural-neurobiological concordance in the effects of CMS. Neuropsychobiology. (2005) 52:90–110. doi: 10.1159/000087097. PMID: 16037678

[70] D'Amico R Gugliandolo E Cordaro M Fusco R Genovese T Peritore AF . Toxic effects of endocrine disruptor exposure on collagen-induced arthritis. Biomolecules. (2022) 12:564. doi: 10.3390/biom12040564. PMID: 35454153 PMC9025575

[71] Basbaum AI Bautista DM Scherrer G Julius D . Cellular and molecular mechanisms of pain. Cell. (2009) 139:267–84. doi: 10.1016/j.cell.2009.09.028. PMID: 19837031 PMC2852643

[72] Dinarello CA . Interleukin-18 and the pathogenesis of inflammatory diseases. Semin Nephrol. (2007) 27:98–114. doi: 10.1016/j.semnephrol.2006.09.013. PMID: 17336692

