# Supplementary material for: Comorbidity risk characteristics of rheumatoid arthritis in the context of depression-associated lipid metabolism
Source: Front Immunol. 2026 May 26;17:1806730. doi: 10.3389/fimmu.2026.1806730 (PMC13246717; doi:10.3389/fimmu.2026.1806730)
Supplement: Supplementary file 1 [file Table1.docx]

Supplementary

Information

***Comorbidity Risk Characteristics of Rheumatoid Arthritis in the Context of Depression-Associated Lipid Metabolism***

**Jiaru Liu^1^, Cuitian Xiang^1^, Qirui Zhou^2^, Zhenzhu Ding^1^, Jingrong Wang^3^, Arong Li^4*^**

*^1^The Second Clinical College of Guangzhou University of Chinese Medicine, Guangzhou, China*

*^2^School of Automation, Guangdong University of Technology, Guangzhou, China*

*^3^State Key Laboratory of Traditional Chinese Medicine Syndrome, the Second Affiliated Hospital of Guangzhou University of Chinese Medicine, Guangzhou, China*

*^4^Chinese Medicine Guangdong Laboratory, Guangdong-Macao In-Depth Cooperation Zone in Hengqin, Zhuhai, China.*

**^*^Correspondence**

Arong Li, Chinese Medicine Guangdong Laboratory, Guangdong-Macao In-Depth Cooperation Zone in Hengqin, Zhuhai, China.

Email: liarong0013@gmail.com

**This PDF file includes:**

Figure S1 to S5

Table S1 to S2


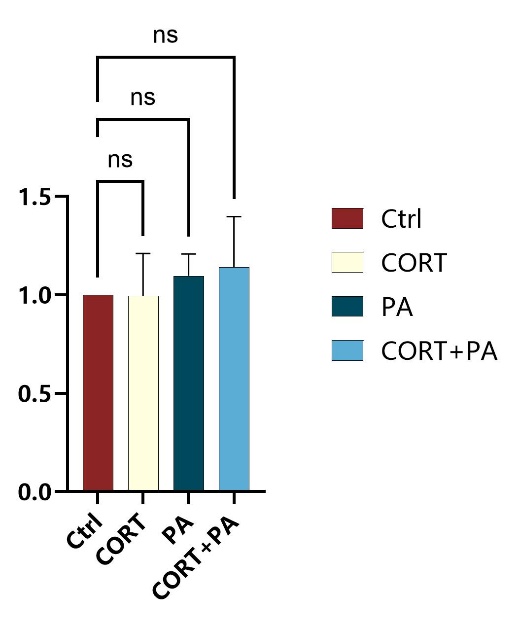


**Figure S1 HT-22 cell viability under different treatment conditions**


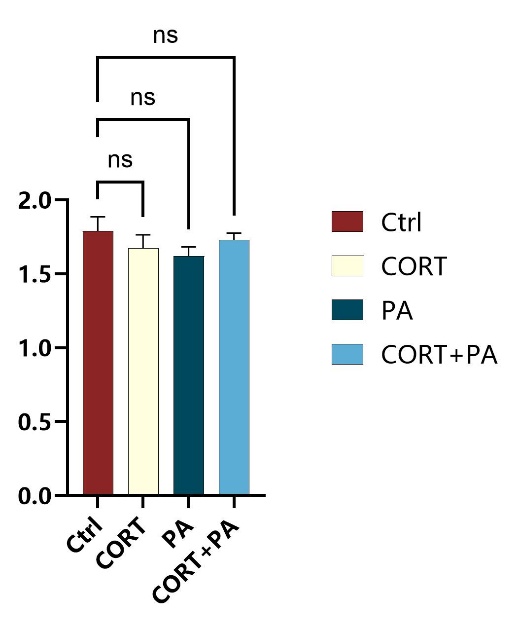


**Figure S2 HT-22 cell numbers under different treatment conditions**


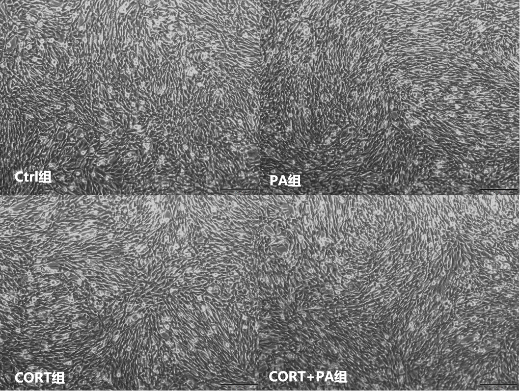


**Figure S3 Representative phase-contrast microscopy images of HT-22 cells under different treatment conditions (10×)**


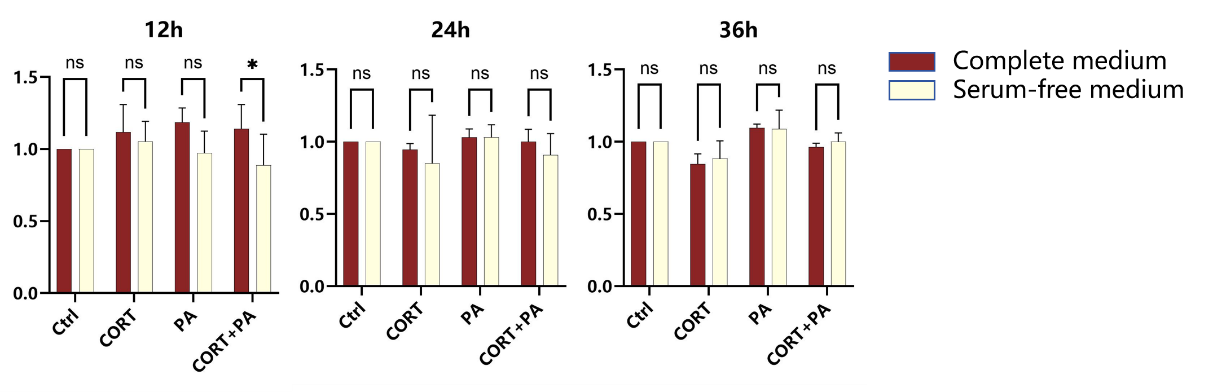


**Figure S4 Cell viability under different serum conditions at 12h, 24h, and 36h**


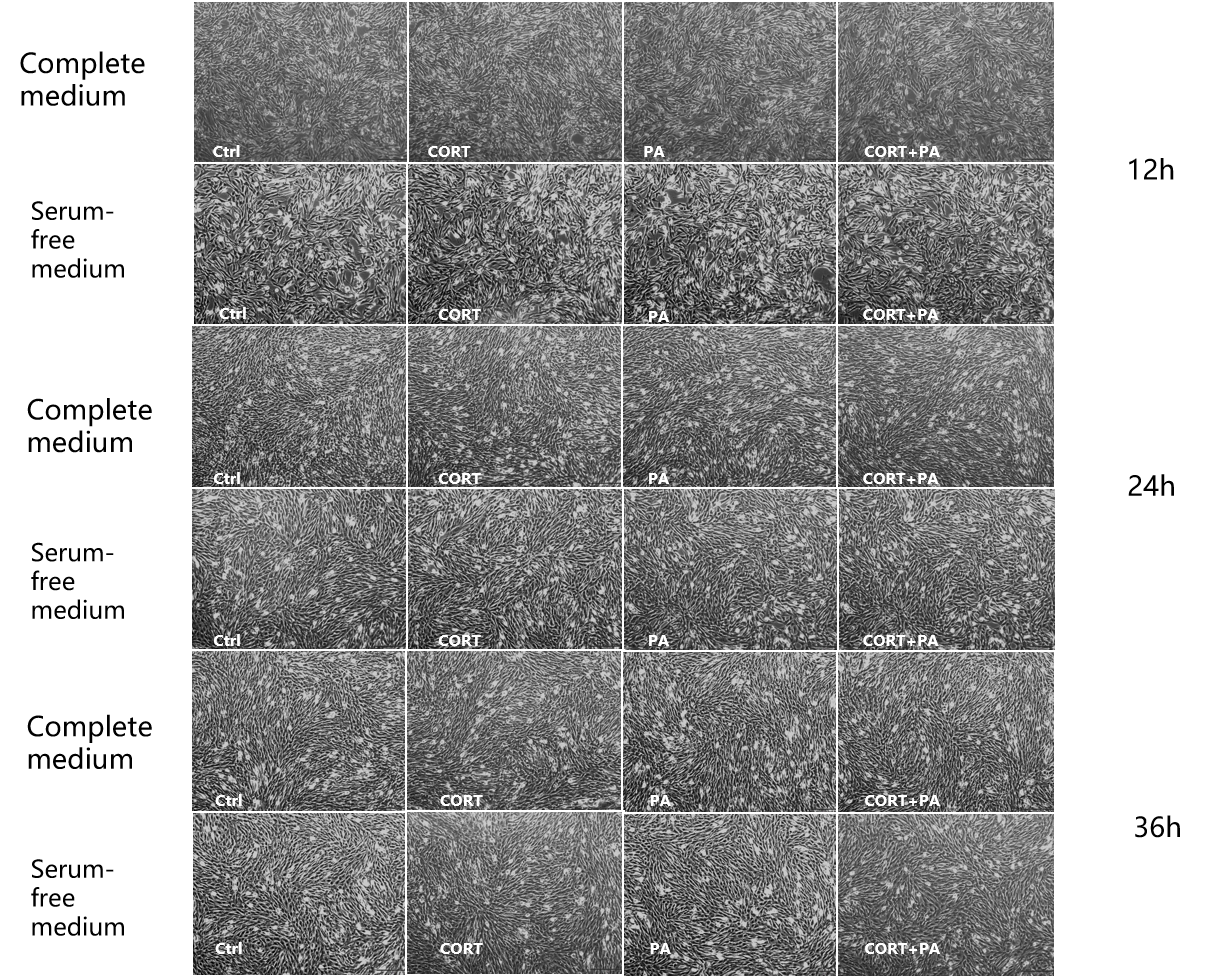


**Figure S5 Cells Morphology under different serum conditions at 12h, 24h, and 36h**

**Table S1 Primer sequences used in this study**

| **Oligo Name** | **Sequence(5' to 3')** | **Length** |
| --- | --- | --- |
| SGK1-F1  SGK1-R1  FKBP5-F1  FKBP5-R1  GRP78-F1  GRP78-R1  CHOP-F1  CHOP-R1  CD59-F1  CD59-R1  TNFSF13B-F1  TNFSF13B-R1  IL18-F1  IL18-R1 | tcc ata agc agc cgt atg acc  ggt gcc ttg ccg agt ttg ta  tat gct tat ggc tcg gct gg  gag tat ccc tcg cct ttc cg  cca atg acc aaa acc gcc tg  att cct ggt gtc aat gcg ct  gga gct gga agc ctg gta tg  atg tgc gtg tga cct ctg t  ctg act gca aaa cag ccg tc  tca ttt tcc ctc aag cgg gt  aag ggg aag tgc cct aga aga  tag atg tcc cat ggc gta ggt  agg aaa tga atc ctc ctg ata aca  agg tct ctc tct ttt tca caa gc | 21  20  20  20  20  20  20  20  20  20  21  21  25  23 |

**Table S2 Correlation of BMI with lipid score, signature score, and gene expression levels in four datasets**

| **dataset** | **score** | **n** | **rho** | ***P* value** | ***FDR*** |
| --- | --- | --- | --- | --- | --- |
| GSE185855 | BMI_MDD_vs_control | 69 | NA | 0.4787 | 0.6266 |
| GSE185855 | lipid_score | 69 | -0.1642 | 0.1775 | 0.2920 |
| GSE185855 | signature_score | 69 | 0.0279 | 0.8197 | 0.9007 |
| GSE185855 | TNFSF13B | 69 | 0.0310 | 0.8005 | 0.8005 |
| GSE185855 | IL18 | 69 | -0.1057 | 0.3872 | 0.7962 |
| GSE185855 | CD59 | 69 | 0.0823 | 0.5014 | 0.6686 |
| GSE251778 | BMI_MDD_vs_control | 169 | NA | 0.6266 | 0.6266 |
| GSE251778 | lipid_score | 169 | 0.0954 | 0.2172 | 0.2920 |
| GSE251778 | signature_score | 169 | 0.1418 | 0.0660 | 0.2639 |
| GSE251778 | TNFSF13B | 169 | 0.0557 | 0.4723 | 0.6298 |
| GSE251778 | IL18 | 169 | 0.0406 | 0.6001 | 0.7962 |
| GSE251778 | CD59 | 169 | 0.1794 | 0.0196 | 0.0784 |
| GSE260603 | BMI_MDD_vs_control | 261 | NA | 0.5680 | 0.6266 |
| GSE260603 | lipid_score | 261 | -0.0433 | 0.4859 | 0.4859 |
| GSE260603 | signature_score | 261 | -0.0078 | 0.9007 | 0.9007 |
| GSE260603 | TNFSF13B | 261 | -0.0770 | 0.2151 | 0.6298 |
| GSE260603 | IL18 | 261 | -0.0161 | 0.7962 | 0.7962 |
| GSE260603 | CD59 | 261 | -0.0936 | 0.1314 | 0.2627 |
| GSE289146 | BMI_MDD_vs_control | 299 | NA | 0.0130 | 0.0518 |
| GSE289146 | lipid_score | 299 | 0.0713 | 0.2190 | 0.2920 |
| GSE289146 | signature_score | 299 | 0.0200 | 0.7310 | 0.9007 |
| GSE289146 | TNFSF13B | 299 | -0.0469 | 0.4186 | 0.6298 |
| GSE289146 | IL18 | 299 | 0.0212 | 0.7154 | 0.7962 |
| GSE289146 | CD59 | 299 | -0.0244 | 0.6744 | 0.6744 |
